# Intraoperative Fast Adaptive Focus Tracking Robotic OCT Enables Real‐Time Tumor Grading and Large‐Area Microvascular Imaging in Human Spinal Cord Surgery

**DOI:** 10.1002/advs.202503566

**Published:** 2025-04-25

**Authors:** Bin He, Yuzhe Ying, Yejiong Shi, Zhe Meng, Zichen Yin, Zhengyu Chen, Zhangwei Hu, Ruizhi Xue, Linkai Jing, Yang Lu, Zhenxing Sun, Weitao Man, Youtu Wu, Dan Lei, Ning Zhang, Guihuai Wang, Ping Xue

**Affiliations:** ^1^ State Key Laboratory of Low‐dimensional Quantum Physics and Department of Physics Tsinghua University Beijing 100084 China; ^2^ Frontier Science Center for Quantum Information Beijing 100084 China; ^3^ Department of Biomedical Engineering College of Design and Engineering National University of Singapore Singapore 117576 Singapore; ^4^ Department of Neurosurgery Beijing Tsinghua Changgung Hospital School of Clinical Medicine and Institute of Precision Medicine Tsinghua University Beijing 102218 China; ^5^ Institute of Forensic Science Ministry of Public Security Beijing 100038 China

**Keywords:** intraoperative imaging, microvascular imaging, Optical coherence tomography, robotic arm, spinal cord tumors, tumor grading

## Abstract

Current surgical procedures for spinal cord tumors lack in vivo high‐resolution multifunctional imaging systems, hindering precise tumor resection. This study introduces the fast adaptive focus tracking robotic optical coherence tomography (FACT‐ROCT) system, which provides real‐time, artifact‐free imaging during surgery, addressing motion artifacts and resolution degradation. Imaging occurs in 22 patients, including 13 with gliomas (WHO grade I‐IV). This represents the first in situ OCT imaging of human spinal cord tumors, enabling the differentiation of tumor types in real‐time. The standard deviation of the attenuation coefficient serves as a physical marker, achieving 90.2%  ±  2.7% accuracy in distinguishing high‐ from low‐grade gliomas intraoperatively at a threshold of 0.75  ±  0.01 mm^−1^. FACT‐ROCT also enables microvascular imaging, covering areas of 70 mm × 13 mm × 10 mm within 2 min and revealing greater vascular tortuosity in higher‐grade tumors. This extensive imaging capability provides critical information that guides surgical strategies, enhancing surgical outcomes. Overall, FACT‐ROCT represents a significant advancement in intraoperative imaging, offering high‐resolution, high‐speed, and comprehensive insights into spinal cord tumor structure and vasculature.

## Graphical abstract

The fast adaptive focus tracking robotic optical coherence tomography (FACT‐ROCT) offers real‐time, high‐resolution imaging of spinal cord tumors during surgery. By reducing motion artifacts with robotics and adaptive focal tracking, it accurately differentiates tumor grades using a novel attenuation coefficient marker and reveals critical microvascular details. FACT‐ROCT represents a major advancement in intraoperative spinal cord imaging techniques.

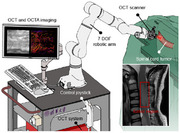

## 1 Introduction

The spinal cord, along with the brain, forms the central nervous system, essential for the body's functioning. The spinal cord is a quasi‐cylindrical structure, ≈10 to 14 millimeters in diameter, running from the brain down through the vertebral column.^[^ ^1^ ^]^ Acting like an optical fiber cable, it transmits signals between the brain and the rest of the body, coordinating motor functions, sensory information, and autonomic responses. Because of its critical role, any abnormal growths, especially tumors, in or around the spinal cord can cause severe neurological impairments. These impairments can result in pain, loss of motor function, sensory deficits, and autonomic dysfunction, significantly affecting a patient's quality of life.^[^ ^2^ ^]^

Surgery is the primary treatment for spinal cord tumors, aiming to remove as much of the tumor as possible while preservingnormal neural structures.^[^ ^3^ ^]^ The spinal cord's limited space and critical functions make this a particularly challenging task, necessitating high‐resolution imaging to accurately identify vital normal structures, such as nerve roots, fasciculus, or grey matter, and avoid damaging them during surgery. Current imaging technologies like MRI,^[^ ^4^ ^]^ CT,^[^ ^5^ ^]^ and ultrasound,^[^ ^6^ ^]^ while beneficial, often fall short in providing the necessary resolution and real‐time capabilities needed for optimal intraoperative guidance. These modalities may struggle with clearly delineating the tumor from surrounding critical normal structures and unable to differentiate tumors between types, which is crucial for effective surgical planning and execution.^[^ ^7^ ^]^ For example, most ependymomas grow slowly and have relatively clear boundaries with normal tissue, allowing for complete resection; however, many astrocytomas exhibit diffuse infiltration, and even complete resection may not necessarily improve the patient's prognosis. Moreover, accurate tumor grading during surgery is also essential for determining the appropriate surgical strategy. High‐grade tumors, such as advanced spinal gliomas, typically require more extensive resection due to their aggressive nature.^[^ ^8^ ^]^ However, the extent of resection must be carefully balanced against the risk of damaging critical neural pathways. Accurate grading helps optimize patient outcomes by tailoring the surgical approach to the tumor's aggressiveness. Currently, intraoperative tumor grading is primarily achieved through frozen section pathology.^[^ ^9^ ^]^ While this method can provide reasonably accurate results (≈70%), it also has limitations, such as being time‐consuming, typically taking around one hour in clinical practice, and the potential for sampling errors, as the small tissue sample may not be representative of the entire tumor.^[^ ^10^ ^]^

Additionally, functional vascular imaging is crucial in spinal cord tumor surgery, as detailed vascular maps allow surgeons to avoid blood vessels during resection, minimizing intraoperative bleeding and improving surgical safety. Moreover, the significant differences in the vascular structures of tumors compared to surrounding healthy tissue are important markers of tumor characteristics.^[^ ^11^ ^]^ International diagnostic standards, such as the WHO,^[^ ^12^ ^]^ Kernohan system,^[^ ^13^ ^]^ and St. Anne/Mayo grading system,^[^ ^14^ ^]^ consider the formation of new blood vessels within the tumor as a key indicator of its malignancy. Understanding the tumor's vascular characteristics also aids in assessing its aggressiveness and planning further treatments. In terms of vascular imaging during spinal cord tumor surgery, techniques such as magnetic resonance angiography (MRA),^[^ ^15^ ^]^ computed tomography angiography (CTA),^[^ ^16^ ^]^ and fluorescence angiography^[^ ^17^ ^]^ are commonly used. MRA and CTA can provide vascular structures with low resolution and lack real‐time application during surgery. While fluorescence angiography, though providing real‐time visualization, is limited by its lack of tomographic capabilities and the need for dye injection. Overall, there remains a significant gap in the availability of a high‐speed, in vivo imaging modality that can effectively differentiate between types and grades of spinal cord tumors and perform in situ vascular imaging during surgery.

Optical coherence tomography (OCT) is an emerging non‐invasive, high‐resolution imaging modality that provides 3D, label‐free visualization of biological tissues, primarily within 1 to 2 mm depths.^[^ ^18^ ^]^ OCT has been widely used in clinical applications, particularly in ophthalmology^[^ ^19^ ^]^ and cardiovascular imaging.^[^ ^20^ ^]^ In recent years, OCT has also been applied to brain tumor imaging.^[^ ^21^ ^]^ Initial studies focused on ex vivo imaging of human brain tumors in both 2D or 3D formats.^[^ ^22^ ^]^ Kut et.al applied OCT to glioma resection surgeries,^[^ ^23^ ^]^ performing in situ imaging of fresh human brain tumor tissues, demonstrating that OCT could differentiate tumor tissue from normal tissue based on attenuation coefficients. However, these studies primarily involved ex vivo tissues, which differ significantly from in vivo tissues in aspects such as blood perfusion. Almasian et al. extended OCT imaging to in vivo human brain tumors,^[^ ^24^ ^]^ but their handheld probe had stability issues, preventing effective vascular imaging and limiting the imaging range. Kuppler et al. integrated OCT systems with surgical microscopes,^[^ ^25^, ^26^ ^]^ achieving in vivo OCT brain tumor images. However, their system had lower resolution and lacked functional vascular imaging. Vakoc et al. applied OCT angiography imaging to mouse brain tumors,^[^ ^27^ ^]^ showing distinctions between tumor and normal tissue vasculature. Nonetheless, they did not extend their research to human brain tumors. Ramakonar et al. further explored in vivo vascular imaging with an OCT probe,^[^ ^28^ ^]^ yet their findings were confined to B‐scan images and lacked volumetric data, thus limiting clinical translation. Overall, OCT still faces several challenges in clinical applications. Intraoperative OCT imaging demands a broader imaging range to cover entire tumor areas. Additionally, to achieve multifunctional in vivo OCT imaging, the imaging system must be highly stable, overcoming motion artifacts caused by living tissues such as heartbeats. Moreover, while OCT has been primarily used for intraoperative imaging of human brain tumors, its application in spinal cord tumor surgery remains unexplored, representing a significant gap in current research.

To address these challenges, we introduce a Fast Adaptive Focus Tracking Robotic Optical Coherence Tomography (FACT‐ROCT) system for intraoperative spinal cord tumor imaging. By mounting the OCT probe on a force‐controlled 7 degrees‐of‐freedom (DOF) robotic arm, we can achieve continuous large‐area automatic imaging, covering the entire spinal cord tumor region safely. To overcome deformation and lateral resolution degradation caused by motion during surgery, we use rapid cross‐sectional B‐scans to acquire surface topology and an electrically tunable lens (ETL) for focus tracking. By utilizing the surface depth information obtained from one B‐scan to drive the focus of the subsequent B‐scan, we achieve a focus tracking speed of ≈10 milliseconds. This enables high‐resolution 3D imaging of various shapes, mitigating resolution degradation due to object motion.

Using the FACT‐ROCT system, we conducted intraoperative OCT imaging on 22 patients with spinal cord tumors, including 13 with spinal gliomas (grades I to IV) and 9 with other tumors or lesions such as vascular reticulocytomas and teratomas. This study marks the first demonstration of OCT in situ imaging of human spinal cord tumors, providing micrometer‐scale in vivo structural images of various tumor types. Our findings reveal the potential of FACT‐ROCT to differentiate between high‐ and low‐grade gliomas with 90.2%  ±  2.7% accuracy in real‐time tumor grading based on attenuation coefficient heterogeneity. Additionally, the FACT‐ROCT system enabled extensive vascular imaging, covering an area of 70 mm × 13 mm × 10 mm in under 2 min. Detailed vascular maps provided critical information for surgical planning, with quantitative comparisons confirming greater vascular tortuosity in higher‐grade tumors. Our study demonstrates the translational potential and practicality of FACT‐ROCT in spinal cord tumor surgery, paving the way for its integration into clinical practice.

## 2 Result

### 2.1 Fast Adaptive Focus Tracking Robotic OCT (FACT‐ROCT) Imaging Approach

**Figure 1:**
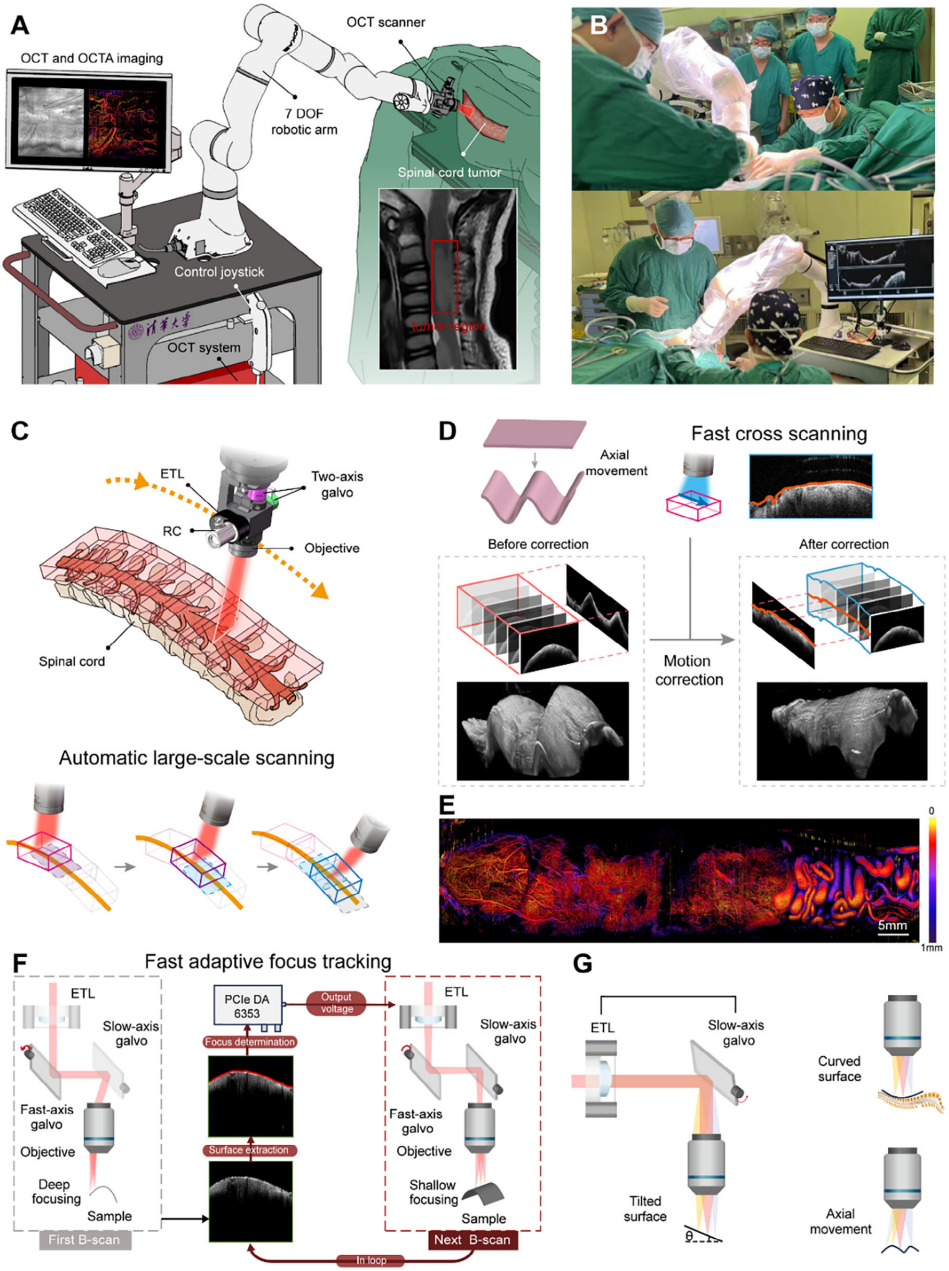
Schematic and Application of the FACT‐ROCT System for Intraoperative Spinal Cord Tumor Imaging. A) Overview of the FACT‐ROCT system setup, which includes an OCT scanner mounted on a 7‐DOF robotic arm. The system performs both OCT and OCT angiography imaging of spinal cord tumors. The inset shows a spinal cord tumor in an MRI scan, indicating the region of interest. B) Photographs demonstrating the use of the FACT‐ROCT system during spinal cord tumor surgery. C) Schematic of the automatic large‐scale scanning mechanism employed by the FACT‐ROCT system. Abbreviations: RC, reflective collimator; ETL, electrically tunable lens. D) Illustration of the fast cross‐sectional scanning and motion correction process. E) The OCTA image of the von Hippel–Lindau (VHL) spinal cord tumor, where the colors indicate different depths. F) Diagram of the fast adaptive focus tracking method. The system uses surface depth information obtained from one B‐scan to drive the focus of the subsequent B‐scan. This process involves a feedback loop for continuous focus refinement. G) Depiction of the FACT‐ROCT's ability to adapt to curved and tilted surfaces, as well as axial movement, maintaining consistent focus and image quality across various anatomical structures.

We conducted a first‐in‐human study using the FACT‐ROCT system on patients undergoing spinal cord tumor resection surgeries. The FACT‐ROCT system employs a probe mounted on a 7‐DOF robotic arm (**Figure** **1**A,C), which features force control capabilities. This robotic arm supports near zero‐force dragging and virtual wall control, ensuring optimal safety during intraoperative imaging. The FACT‐ROCT system is based on a customized swept‐source OCT system that utilizes a high‐speed (200 kHz) near‐infrared (1310 nm) laser, providing a long coherence length and high axial resolution of ≈10 µm within the tissue. This system achieves 3D structural and vascular imaging of the tissue by scanning with fast and slow‐axis galvo mirrors, and collecting the backscattered light from the different layers of the tissue, as detailed in the Experimental Section.

Prior to imaging, the surgeon uses MRI images to determine the approximate location of the tumor. The surgical procedure involves making an incision through the skin and muscles, removing the lamina, and opening the dura mater to expose the spine and tumor (Figure 1B). These steps are standard preoperative procedures for spinal cord tumor resection surgery. For safety, the surgeon first uses real‐time OCT cross‐sectional images to manually guide the probe to the leading edge of the spinal cord tumor with the robotic arm. Once the initial OCT volume is obtained, the robotic arm automatically moves to the next imaging position based on the previous OCT volume (detailed in the Experimental Section), enabling extensive imaging of the entire spinal cord tumor region (Figure 1C). The FACT‐ROCT system can acquire 280 cross‐sectional images per second and displaying them in real‐time. It enables volumetric structural imaging within 2.5 s and volumetric vascular imaging within 10 s. The single scan range of the system reaches 13 mm × 13 mm × 5 mm, covering the diameter of the spinal cord and allowing for extensive lesion coverage through the movement of the robotic arm.

During intraoperative OCT imaging, tissue movement caused by the patient's heartbeat is inevitable, leading to two significant issues. First, it causes severe deformation of the OCT volume, manifesting as a hump‐like distortion, as shown in Figure 1D. To solve this issue, we perform a fast cross‐sectional scan using the original slow‐axis galvo mirrors before each OCT volumetric imaging session. This approach captures the object's original contour along the slow axis, as indicated by the red line in Figure 1D, allowing for the correction of the object's original 3D shape with minimal added imaging time.

Additionally, heartbeat‐induced jitter can also cause frequent defocusing of the imaging plane. To solve this, we propose a novel rapid adaptive focus tracking technique to maintain focus during imaging. It involves a feedback loop for continuous focus refinement without needing prior knowledge of the object's shape. Specifically, an ETL is integrated into the OCT probe. The depth information obtained from the previous B‐scan can drive the ETL to achieve the accurate focus of the next B‐scan, thereby enabling almost all B‐scans to be focused (Figure 1F), achieving focus tracking speeds of ≈10 ms. The capability of fast focus tracking makes the FACT‐ROCT suitable for tissues with curved and tilted surfaces in addition to the tissue movement, it could maintain consistent focus and image quality across various anatomical structures (Figure 1G). In addition to OCT structural imaging, the OCT angiography image of a VHL spinal cord tumor, shown in Figure 1E, demonstrates the capability of FACT‐ROCT in achieving large‐scale, high‐resolution intraoperative vascular imaging, while also revealing the high heterogeneity of the tumor's vasculature. Unlike traditional ETL‐based OCT,^[^ ^29^, ^30^, ^31^ ^]^ which requires multiple volumetric scans or pre‐scans to maintain high resolution for complex curved objects, thereby increasing imaging time, FACT‐ROCT can achieve high‐resolution imaging of the entire curved object with a single volumetric scan‐based on real‐time focus tracking. Additionally, this method does not suffer from sensitivity loss like special beams such as needle beams or Bessel beams,^[^ ^32^, ^33^ ^]^ and it can be applied to any OCT system, demonstrating great versatility.

### 2.2 Focus Tracking Performance of the FACT‐ROCT System

**Figure 2:**
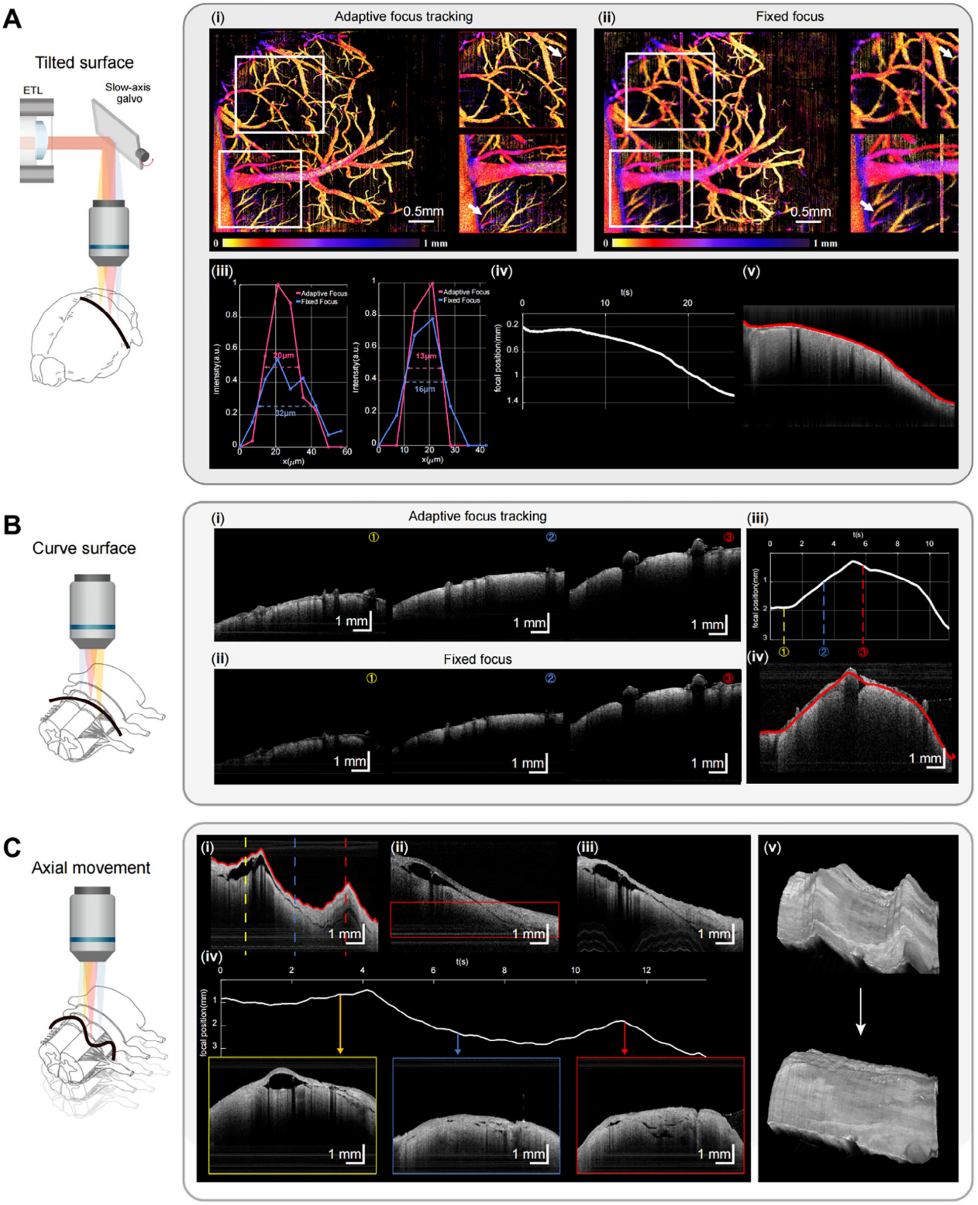
Evaluation of the focus‐tracking capability of the FACT‐ROCT System. A) Imaging of a tilted mouse brain, comparing OCT angiography using the adaptive focus tracking method (i) and the fixed focus method (ii). The entire region in panel (i) shows clear vascular imaging, whereas in panel (ii), the left half appears blurry. Insets highlight the regions of interest. iii) Quantitative analysis of vessel diameter under adaptive focus (red line) versus fixed focus (blue line). iv) Focus position correction curves over time, corresponding to surface profiles along the slow axis of the OCT volume (v). B) Imaging of a curved human spinal cord, showing OCT structural images using the adaptive focus tracking method (i) and the fixed focus method (ii). iii) Focus position correction curves over time, corresponding to surface profiles along the slow axis of the OCT volume (iv). C) Imaging of a human spinal cord with significant axial movement: i) OCT B‐scan along the slow axis of the galvanometer, showing longitudinal jitter. ii) OCT B‐scan along the slow axis under fast cross‐scanning, showing no motion artifacts, though only the region within the red box remains within the depth of focus. iii) OCT B‐scan along the slow axis after motion compensation, showing artifact removal and all regions within the depth of focus. iv) Focus position correction curves over time for adaptive focusing, indicating an axial shift of ≈3 mm. OCT B‐scan images at the arrowed positions confirm all regions within the depth of focus, with high signal‐to‐noise ratios. v) OCT volume images before and after motion compensation.

We further assess the focus tracking capability of the FACT‐ROCT system on tissues with various shapes and axial movement, utilizing both high lateral resolution (8 µm) and low lateral resolution (25 µm) objectives to demonstrate its versatility, as shown in **Figure** **2**. For high lateral resolution imaging, we conducted OCT angiography on a tilted mouse brain to evaluate vascular clarity. The mouse brain's cortical curvature, with a depth variation exceeding 1 mm, limits traditional fixed‐focus approaches, resulting in a focused area confined to a small region and blurriness elsewhere. In contrast, the FACT‐ROCT, with continuous feedback‐based focus adjustments, achieved uniform vascular imaging across all positions by applying the focal correction curve (Figure 2A‐iv). As a result, the adaptive focus method (Figure 2A‐i) provides clear vascular imaging across the entire region, while the fixed focus method (Figure 2A‐ii) leaves the left side blurred. Quantitative vessel diameter analysis (Figure 2A‐iii) confirms this difference, showing up to a 60% improvement in resolution with the adaptive focus technique, highlighting FACT‐ROCT's advantages in angiographic imaging.

For structural imaging, we compared the performance of FACT‐ROCT and traditional OCT on a curved human spinal cord, using low lateral resolution imaging for a larger field of view (13 mm × 13 mm). The significant curvature of the spinal cord, with ≈a 2.5 mm depth variation, affects traditional OCT images, causing blurring in peripheral regions (Figure 2B‐ii). In contrast, with adaptive focus tracking, the FACT‐ROCT achieves uniformly sharp structural imaging across the entire field (Figure 2B‐i), maintaining high SNR and detail. The focal position correction curve (Figure 2B‐iii) demonstrates real‐time adjustments corresponding to the surface profile of the spinal cord (Figure 2B‐iv), further underscoring the FACT‐ROCT's adaptability in handling complex shapes.

Last, we assessed the FACT‐ROCT's ability to maintain focus under significant axial movement, a condition typical in intraoperative imaging. With ≈3 mm of axial shift, the OCT B‐scan along the slow axis without compensation shows pronounced jitter artifacts (Figure 2C‐i). While fast cross‐scanning (Figure 2C‐ii) eliminates motion artifacts, it restricts the in‐focus region to a small area (highlighted in red). After applying motion compensation and focus tracking, the FACT‐ROCT removes artifacts and maintains focus across the entire field of view (Figure 2C‐iii). The focus correction curves over time (Figure 2C‐iv) reflect the system's adaptive responses, with OCT B‐scans at multiple points showing consistently high SNR and depth focus. Volume images (Figure 2C‐v) before and after compensation demonstrate the FACT‐ROCT's effectiveness of motion correction.

### 2.3 Intraoperative Imaging of Spinal Cord Tumors with FACT‐ROCT

We have utilized FACT‐ROCT to perform intraoperative spinal tumor imaging on 22 patients, serving as a complementary imaging modality alongside the surgical microscope to assess tumor characteristics. During imaging, the system achieves an axial resolution of 10 µm and a lateral resolution of 25 µm. The 1310 nm wavelength enables a penetration depth of 1 to 2 mm for spinal cord tumor imaging. The patient cohort, detailed in Table S2 (Supporting Information), primarily included spinal cord gliomas such as ependymomas and diffuse midline gliomas (DMGs), as well as other tumors like hemangiomas and teratomas. Most cases presented preoperatively with motor weakness and sensory disturbances, while a minority exhibited bowel/bladder dysfunction. Following FACT‐ROCT‐guided surgery, most patients demonstrated early postoperative symptom improvement without new neurological deficits or surgery‐related complications. A small subset experienced transient symptom exacerbation, but most of them recovered to baseline functional status within several months to one year of follow‐up. All patients underwent postoperative MRI within 72 h, with the extent of resection classified as: gross total resection (GTR), subtotal resection (STR, <20% residual tumor volume), or partial resection (PR, ≥20% residual). Our results showed that under FACT‐ROCT guidance, 18 cases achieved GTR, while only 3 cases were STR and 1 case PR. Intraoperative blood loss remained within acceptable limits for all cases (mean: 122.38 ml; range: 20–400 ml), demonstrating both the safety profile and high resection rates achievable with FACT‐ROCT guidance, without significantly prolonging operative duration. During follow‐up, two patients expired: Case 10 succumbed to rapidly progressive disease (DMG, H3K27M+) that proved refractory to reoperation and adjuvant therapy, while Case 18 died from intracranial recurrence and disseminated spinal metastases of malignant meningioma. The remaining patients showed no evidence of recurrence during follow‐up, though the relatively short observation period and limited sample size necessitate longer‐term surveillance. For each patient, multiple scans were performed using the robotic arm to achieve complete coverage of the tumor area, with all imaging mostly completed within 2 min. Data was mostly collected both before and after tumor resection. Additionally, given that OCT is a non‐contact, label‐free, and non‐destructive imaging technique, FACT‐ROCT did not cause any imaging‐related side effects, and no adverse events associated with OCT use were observed, underscoring its safety and tolerability in clinical practice.

**Figure 3:**
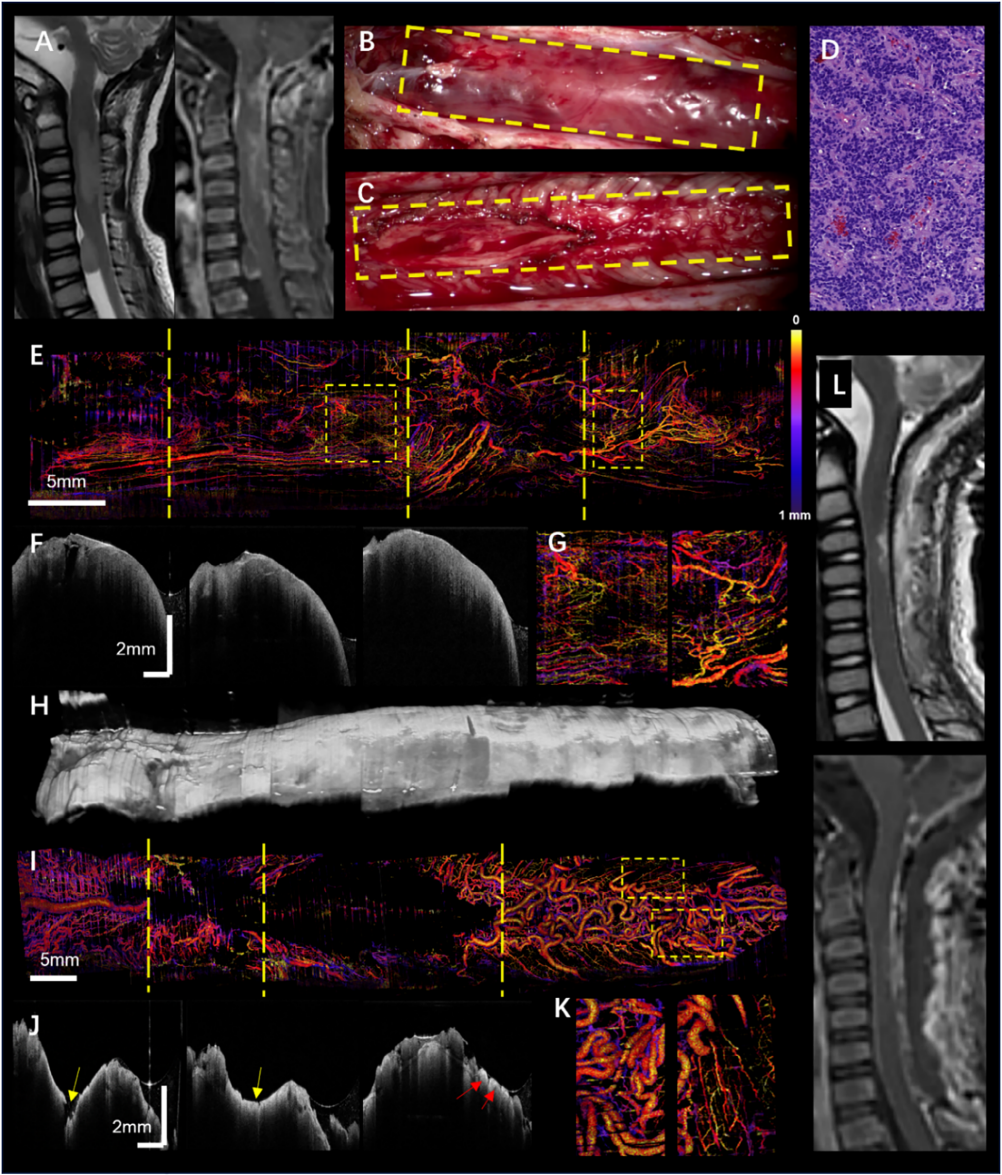
A case of recurrent ependymoma with FACT‐ROCT: A) Preoperative MRI shows the tumor in the cervical spinal canal, causing fusiform swelling and surface breakthrough. Contrast‐enhanced MRI reveals heterogeneous signals. B) Intraoperative microscopic image of the tumor, located on the spinal cord surface with a rich blood supply, though surface vasculature appears sparse. C) Post‐tumor resection, showing the surgical cavity. D) Postoperative pathology shows moderately dense tumor cells with moderate atypia, forming rosettes and perivascular pseudorosettes, characteristic of WHO Grade III ependymoma. E) OCT angiography shows dense and irregular tumor vasculature, with large surface vessels corresponding to panel B. F) Selected OCT B‐scan images from the tumor's rostral to caudal ends, displaying heterogeneous scattering signals. G) Magnified vascular image from the yellow dashed box in panel E, showing dense and heterogeneous tumor vasculature. H) A 3D OCT structural image of the entire tumor region shows its 3D morphology. I) Post‐resection OCTA image reveals no vascular signals within the surgical cavity, with surface vessels corresponding to panel C. J) Selected post‐resection OCT B‐scan images shows uniform optical signals at the tumor bed (yellow arrows). Blood vessels and nerve roots are observed on the caudal surface (red arrows) K) Magnified vascular image from the yellow dashed box in panel E. L) Postoperative MRI shows no residual tumor.

Here, we present a detailed case to demonstrate the utility of OCT and OCT angiography (OCTA) in intraoperative spinal tumor imaging. The patient, a 4‐year‐old male, had undergone intracranial ependymoma resection three years ago. Preoperative MRI (**Figure** **3**A) revealed two intramedullary lesions in the cervical spinal canal, the larger measuring ≈59 × 10 mm, with significant and heterogeneous signals upon contrast‐enhanced MRI. During the surgery, after incising the dura mater and suspending it, the tumor was fully exposed. The tumor extended from the fourth ventricular outlet to the C7 vertebral level, appearing soft, grayish‐red, and lobulated, suggesting a rich blood supply. However, the surgical microscope could hardly visualize the microvasculature (Figure 3B). After partial tumor resection, it was observed that parts of the tumor had invaded the spinal cord parenchyma at the C3‐C5 vertebral levels. Complete tumor resection was achieved, and the surgical field at the C3‐C7 vertebral levels post‐resection is shown (Figure 3C). Postoperative pathological examination (Figure 3D) revealed highly dense tumor cells with moderate atypia, forming rosettes and perivascular pseudorosettes, consistent with WHO Grade III ependymoma.

Following tumor exposure, we employed FACT‐ROCT to image the entire tumor region. The OCT angiography image (Figure 3E) revealed a rich and heterogeneous blood supply within the tumor. The magnified region of interest (Figure 3G) further demonstrated the high density and tortuosity of the tumor, consistent with the characteristics of WHO Grade III ependymoma microvascular proliferation. This characteristic of tumor vessels suggests that vascular characteristics could serve as markers for tumor grading. Additionally, the OCT angiography results of this patient revealed an uneven distribution of vascular density within the tumor region, with the left side showing a lower vascular density compared to the right side. This feature could guide strategies for safe tumor resection during surgery. OCT B‐scan structural images (Figure 3F) showed heterogeneous scattering signals within the tumor, indicative of significant tumor heterogeneity, consistent with high‐grade malignancy. And the 3D OCT structural images (Figure 3H) depicted the tumor's 3D morphology. Post‐resection OCT angiography images (Figure 3I,K) showed vascular occlusion and disrupted blood supply in the tumor‐invaded areas due to bipolar coagulation, while surface vasculature was preserved in non‐invaded spinal cord regions. Post‐resection OCT structural images (Figure 3J) demonstrated uniform optical signals within the spinal cord parenchyma at the tumor bed (indicated by yellow arrows). Some blood vessels and nerve roots were observed on the caudal surface of the surgical area (indicated by red arrows). Follow‐up MRI (Figure 3L) confirmed the absence of a residual tumor. This case illustrates the role of FACT‐ROCT in enhancing intraoperative visualization of tumor vasculature and structure, facilitating precise and safe tumor resection, and ensuring postoperative assessment. Additional cases, such as diffuse midline glioma and subependymal tumor, are presented in the Supporting Information in Figures S2,S3 (Supporting Information).

### 2.4 Comparative Imaging of Normal and Tumor Tissues Using FACT‐ROCT

**Figure 4:**
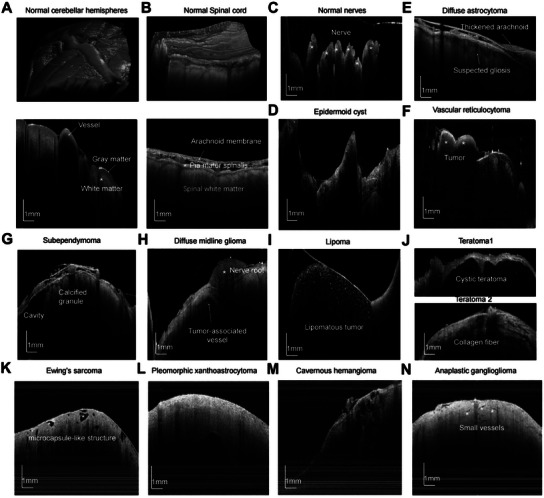
Images of normal tissues and different spinal cord tumors obtained by FACT‐ROCT. A) 3D OCT structural image of the normal human cerebellar hemisphere and one OCT B‐scan image, highlighting a clear boundary between the upper gray matter and lower white matter. B) 3D OCT structural image of the normal human spinal cord and one OCT B‐scan cross‐section, showing a uniform and continuous dark band between the spinal cord white matter and pia mater, with small blood vessels visible in the subarachnoid space. C) OCT image of normal nerve roots, with nerve roots float in the cerebrospinal fluid. D) OCT image of an epidermoid cyst, with cyst contents below the cyst wall showing mixed scattering signals. E) Image of diffuse astrocytoma with suspected gliosis visible. F) OCT image of a vascular reticulocytoma with an irregular shape located at the normal medullary‐cervical junction. G) Image of a subependymal tumor showing features such as cavities and calcified granules. H) OCT image of a DMG showing a nerve root located on the tumor surface and tumor‐associated abnormal vessels. I) OCT image of a lipoma with hyperintense fat granules. J) OCT images of teratomas, with one image showing cyst and the other showing collagen fibers. K) OCT images of ewing's sarcoma with the feature of microcapsule‐like structure. L) OCT images of pleomorphic xanthoastrocytoma with an irregular cystic change in the deep part of the tumor. M) OCT images of cavernous hemangioma showing the feature of Spongiform changes. N) OCT images of Anaplastic ganglioglioma with small blood vessels passing through the tumor.

In this section, FACT‐ROCT was utilized to obtain detailed imaging of normal tissues and various spinal cord tumors. The images provided critical insights into the structural differences between these tissues, suggesting that FACT‐ROCT has the potential in distinguishing different types of spinal cord tumors. For instance, we imaged a normal human cerebellar hemisphere and spinal cord individually. The 3D OCT structural image of the cerebellar hemisphere (**Figure** **4**A) clearly highlighted the boundary between the upper gray matter and the lower white matter. In contrast, the 3D OCT image of the normal human spinal cord (Figure 4B) shows a different structure, with the white matter located above the gray matter. Additionally, there was a uniform and continuous dark band between the spinal cord white matter and the pia mater, with small blood vessels visible in the subarachnoid space. The OCT image of normal nerve roots floating in the cerebrospinal fluid (Figure 4C) further demonstrated that FACT‐ROCT was capable of identifying vital structures that were critical during surgery.

Moving to pathological tissues, OCT images provide some insights into different tumor types. The OCT image of the epidermoid cyst (Figure 4D) shows that the cyst contents are below the cyst wall and exhibit mixed scattering signals, indicating various tissue compositions. In the case of diffuse astrocytoma (Figure 4E), suspected gliosis is evident (marked with a red dashed box). The vascular reticulocytoma (Figure 4F) is seen as an irregularly shaped mass at the bulbar cervical medullary junction. The OCT image of the subependymal tumor (Figure 4G) reveals typical characteristic features such as cavities and calcified granules. Additionally, an OCT image of a DMG (Figure 4H) shows a nerve root located on the tumor surface along with tumor‐associated abnormal vessels, emphasizing the complex vascular structures that can be visualized. A lipoma with hyperintense fat granules is clearly illustrated in an OCT image (Figure 4I), while Figures 4J present OCT images of teratomas, one showing a cyst and the other displaying collagen fibers. Besides, Figure 4K–N shows OCT images of Ewing's sarcoma, Pleomorphic xanthoastrocytoma, Cavernous hemangioma, Anaplastic ganglioglioma with their own unique features. These findings underscore the potential of FACT‐ROCT in providing high‐resolution images that differentiate between different types of spinal cord tumors, thereby facilitating effective intraoperative and postoperative assessments.

### 2.5 Analysis of OCT Structural Images for Spinal Cord Gliomas with Different Grades

We further utilized the FACT‐ROCT system for intraoperative imaging of spinal cord gliomas of various grades, involving two patients with Grade I, four with Grade II, three with Grade III, and four with Grade IV tumors. In vivo OCT structural images were acquired during these procedures, with imaged areas selected based on MRI indications of tumor parenchyma and tumor grades were confirmed by postoperative pathological diagnosis.

**Figure 5:**
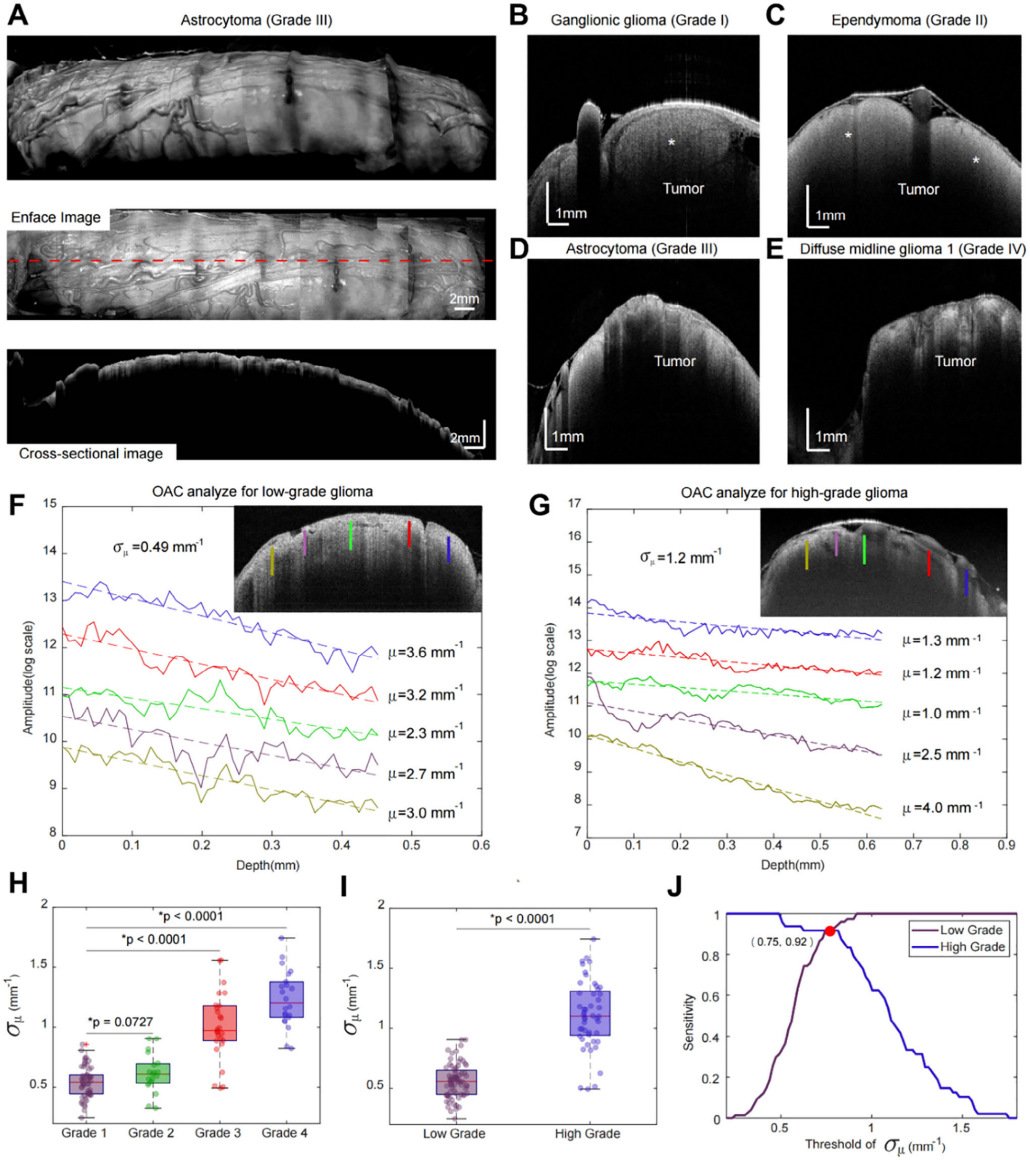
FACT‐ROCT Structural Images and Standard Deviation Analysis of OAC for Different Grades of Spinal Cord Gliomas. A) 3D OCT structural image, enface image, and cross‐sectional image of an Astrocytoma (Grade III), the red dashed line marks the position of the cross‐sectional image. B–E) the typical OCT B‐scan images of Spinal Cord Gliomas with Different Grades. F,G) Depth‐dependent A‐line intensity profiles for low‐grade and high‐grade gliomas at different locations, with each profile averaged over 10 adjacent A‐lines and corrected for focal depth effects, shown in logarithmic scale. The calculated attenuation coefficients are obtained from fitted curves. Different colors indicate A‐lines from various locations, as shown by the corresponding colors in the upper right B‐scan image. For visualization purposes, *y*‐axis offsets of 0, 0.5, 1, 2.5, and 4 have been applied. Here, 𝜇 represents the attenuation coefficient, and 𝜎_𝜇_ represents the standard deviation of the attenuation coefficient. H) Box plot showing the distribution of the standard variance of OAC extracted from different B‐scans (*n* = 122) for different grades of gliomas, showing that the standard deviation of OAC increases with higher grades. I) Box plot comparing the standard variance of OAC (*n* = 122) between low‐grade (Grade I and II) and high‐grade (Grade III and IV) gliomas. J) Sensitivity analysis of standard variance of OAC threshold values for distinguishing between low‐grade and high‐grade gliomas, achieving 90.2%  ±  2.7% accuracy at the threshold of 0.75  ±  0.01 mm^−1^. For all box plots, the center lines represent the median values. The boxes extend from the lower quartile to the upper quartile, and the whiskers represent 1.5 times the interquartile range.

The detailed 3D OCT structural images, en‐face images, and cross‐sectional images of an Astrocytoma (Grade III) (**Figure** **5**A) demonstrate FACT‐ROCT's capability to capture a large tumor region, covering an area of 60 mm × 13 mm × 10 mm. Additionally, the cross‐sectional OCT structural images of tumors revealed that higher‐grade tumors exhibit more uneven scattering signals. The typical OCT B‐scan images (Figure 5B–E) further depicted differences in tissue structure or composition among spinal cord gliomas of different grades. Typical OCT B‐scan images for different grades of spinal cord gliomas (Figure 5B–E) further illustrate variations in tissue structure and composition across grades. To quantitatively analyze the differences in OCT images between grades, we developed a method to obtain the optical attenuation coefficient (OAC) of the tumors, eliminating the depth‐dependent effects of the beam profiles. Since FACT‐ROCT consistently ensures the focus remains at a fixed position within the sample, we can obtain reliable OAC measurements across measurements (See Experimental Section for more details). Unlike previous studies on ex vivo human brain gliomas, we found significant heterogeneity in the OACs of in vivo spinal cord gliomas, with overlapping OAC values across different grades, making it difficult to distinguish tumor grades solely based on OAC. However, we observed notable differences in the variance of the OAC within a single B‐scan across different tumor grades, which aligns with the understanding that tumor heterogeneity increases with higher grades.

We presented two examples of OAC analysis within a single B‐scan for different grades of spinal cord gliomas, with depth‐dependent A‐line intensity profiles for low‐grade and high‐grade gliomas shown in Figure 5F,G. Each profile, averaged over 10 adjacent A‐lines and corrected for focal depth effects, is displayed on a logarithmic scale, with attenuation coefficients derived from fitted curves. For low‐grade gliomas (Figure 5F), the calculated attenuation coefficients (𝜇) range from 2.3 to 3.6 mm⁻¹, with a standard deviation (𝜎_𝜇_) of 0.49 mm⁻¹. In contrast, for high‐grade gliomas (Figure 5G), the attenuation coefficients (𝜇) vary more widely, from 1 to 4 mm⁻¹, with a significantly higher standard deviation (𝜎_𝜇_) of 1.2 mm⁻¹, reflecting the increased heterogeneity associated with higher‐grade tumors.

Additionally, we performed statistical analysis on the standard deviation of attenuation coefficients for all spinal cord glioma data (see Experimental Section for more details). The box plots (Figure 5H) highlight the distribution of the standard variance of OAC, showing a clear increase in the standard deviation of OAC with higher tumor grades. This suggests that the optical properties of gliomas become more variable as the grade increases, reflecting greater heterogeneity within the tumor tissues. Furthermore, the comparison of the standard variance of OAC between low‐grade (Grade I and II) and high‐grade (Grade III and IV) gliomas (Figure 5I) underscores the significant differences in optical characteristics between these two categories. The sensitivity analysis (Figure 5J) reveals that a threshold value of 0.75  ±  0.01 mm^−1^ for the standard variance of OAC can distinguish between low‐grade and high‐grade gliomas with over 90.2%  ±  2.7% accuracy. This high level of accuracy demonstrates the potential of FACT‐ROCT in providing real‐time tumor grading during surgery, which is crucial for determining the appropriate surgical strategy for spinal cord gliomas. We also collected the Ki‐67 proliferation index and the tumor marker H3K27 M as biological assay indicators and compared these with ROCT imaging results, as shown in Figures S7,S8 (Supporting Information). We found a positive correlation between ROCT imaging results and the Ki‐67 proliferation index, with a correlation coefficient of *r* = 0.67, indicating that higher OAC variability is associated with increased tumor proliferation. Additionally, we observed significant differences in ROCT results between groups of patients with different H3K27 M test outcomes, suggesting that while ROCT can differentiate between H3K27 M status. These results elucidate the potential mechanisms underlying the differences in OAC variability among tumors of different grades at a microscopic level, further supporting our conclusions.

### 2.6 OCT Angiography Images for Spinal Cord Gliomas with Different Grades

**Figure 6:**
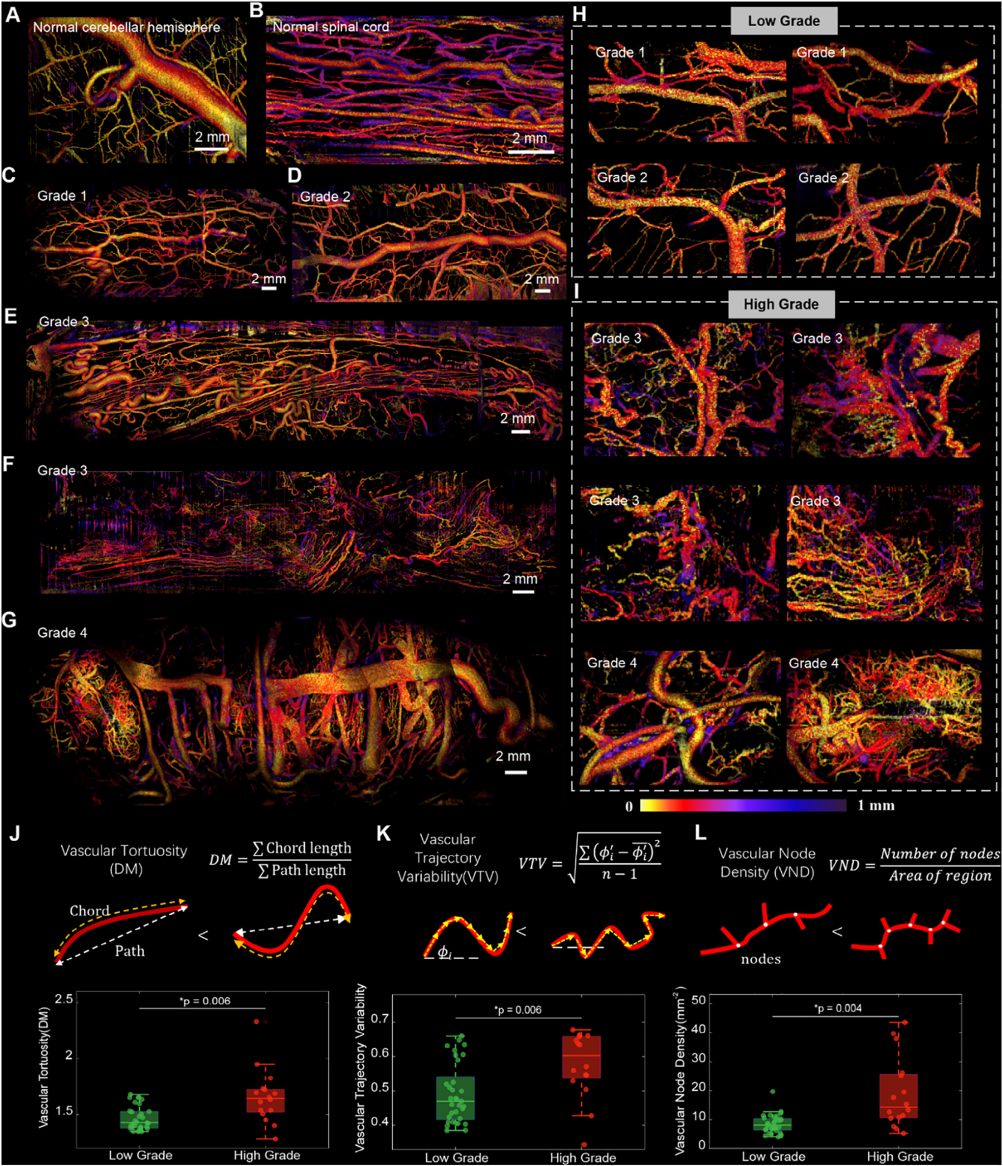
FACT‐ROCT angiography results for the normal tissue and Spinal Cord Gliomas with Different Grades. A) The OCTA image of a normal cerebellar hemisphere reveals prominent draining vessels alongside smaller vessels. B) The OCTA image of the normal thoracic spinal cord displays a structured vascular network with vessels oriented along its long axis, highlighting the organized nature of healthy spinal cord vasculature. C,D) OCTA images of Grade I and Grade II spinal cord gliomas, respectively. illustrate relatively regular and moderately dense surface vasculature, characteristic of low‐grade gliomas. E,F) The OCTA image of a WHO Grade III spinal cord glioma highlights an increased density and heterogeneity of the surface vasculature compared to lower‐grade tumors, suggesting higher angiogenic activity. G) The OCTA image of a WHO Grade IV spinal cord glioma shows a markedly dense and heterogeneous vascular network, with extensive microvascular proliferation and a mix of large draining vessels and irregular small vessels, indicative of high‐grade gliomas. H) Enlarged OCTA images of low‐grade gliomas (Grade I and II) reveal moderately dense, regular vasculature with low heterogeneity, reinforcing the less aggressive nature of these tumors. I) Enlarged OCTA images of high‐grade gliomas (Grade III and IV) display dense, irregular vasculature with significant heterogeneity and pronounced microvascular proliferation, reflecting the aggressive angiogenic profile of high‐grade tumors. J–L) Quantitative analysis of vascular complexity in high‐ and low‐grade spinal cord gliomas, including vascular tortuosity, vascular trajectory, variability, and vascular node density. Each vascular parameter value is derived from an independent subregion of OCTA images (*n* = 49). High‐grade gliomas show higher values with significant differences (*p* < 0.006).

We also utilized FACT‐ROCT angiography to investigate the vascular characteristics of normal tissues and spinal cord gliomas of various grades. The OCTA images provide insightful comparisons between healthy and tumorous tissues, emphasizing the variations in vascular structures associated with different tumor grades. In normal tissues, the cerebellar hemisphere (**Figure** **6**A) exhibits prominent draining vessels alongside smaller vessels, while the thoracic spinal cord (Figure 6B) shows a structured vascular network oriented along its long axis, indicating organized vasculature. Low‐grade gliomas, represented by Grade I (Figure 6C) and Grade II (Figure 6D), display relatively regular and moderately dense surface vasculature, characteristic of their less aggressive nature. In contrast, the OCTA images of high‐grade gliomas, such as Grade III (Figure 6E,F) and Grade IV (Figure 6G), reveal significantly increased density and heterogeneity in the vascular network. Grade III gliomas show a higher density and varied surface vasculature, indicating elevated angiogenic activity, whereas Grade IV gliomas demonstrate a markedly dense and heterogeneous network, with extensive microvascular proliferation and a mix of large draining vessels and irregular small vessels, reflecting the aggressive nature of these tumors. Enlarged images of low‐grade gliomas (Figure 6H) further emphasize the moderately dense, regular vasculature with low heterogeneity, reinforcing their less aggressive behavior. Conversely, high‐grade gliomas (Figure 6I) present dense, irregular vasculature with significant heterogeneity and pronounced microvascular proliferation, highlighting their aggressive angiogenic profile.

Quantitative analyses of vascular complexity parameters further support these visual observations (See Experimental Section for more details). The vascular tortuosity (Figure 6J), vascular trajectory variability (Figure 6K), and vascular node density (Figure 6L) metrics all indicate significantly higher complexity in high‐grade gliomas compared to low‐grade gliomas, with statistically significant differences (*p* = 0.006 for tortuosity and trajectory variability, and *p* = 0.004 for node density). This quantitative evidence underscores the potential of FACT‐ROCT angiography in distinguishing between low‐grade and high‐grade spinal cord gliomas based on their vascular characteristics.

## 3 Discussion

In this study, we present a first‐in‐human study of the multifunctional Optical Coherence Tomography technology, FACT‐ROCT, for intraoperative imaging of spinal cord tumors. Spinal cord tumors pose a substantial clinical challenge due to their location and the critical functions of the spinal cord. Traditional intraoperative imaging modalities such as MRI, CT, and ultrasound, although valuable, often lack the necessary real‐time, high‐resolution capabilities required for optimal intraoperative guidance. FACT‐ROCT addresses these limitations by providing superior spatial resolution, which is approximately an order of magnitude higher than MRI, CT, ultrasound, and intraoperative fluorescence imaging while maintaining real‐time imaging capabilities. Although the resolution of OCT is lower than that of confocal endomicroscopy,^[^ ^34^ ^]^ it achieves significantly greater imaging depth, reaching 1–2 mm compared to the shallow 100–200 µm depth of confocal endomicroscopy. This combination of high‐resolution, label‐free imaging with moderate imaging depth makes FACT‐ROCT a powerful complementary tool for real‐time intraoperative imaging. A detailed comparison of FACT‐ROCT with other intraoperative imaging modalities across parameters such as spatial resolution, imaging depth, exogenous labeling requirements, real‐time capability, and clinical use is provided in Table S1 (Supporting Information). FACT‐ROCT has demonstrated artifact‐free, high‐resolution imaging of various types of spinal cord tumors, capturing micrometer structural and vascular information essential for surgical decision‐making. The system has successfully imaged different grades of spinal cord tumors, including Grade I through Grade IV gliomas, providing clear differentiation between tumor grades based on structural and vascular characteristics.

Our FACT‐ROCT system incorporates several key innovations pivotal for its success in a clinical setting. First, the rapid adaptive focus tracking method, achieved through the integration of an ETL, ensures that the imaging focus is consistently maintained despite tissue movements. This real‐time focus adjustment, with a response time of ≈10 ms, allows for high SNR and high‐resolution imaging across varying tissue surfaces. Unlike traditional ETL‐based OCT techniques that require multi‐volume scanning or pre‐scanning,^[^ ^29^, ^30^, ^31^ ^]^ our method involves a feedback loop for continuous focus refinement without needing prior knowledge of the object's shape. Therefore, FACT‐ROCT is also able to addresses the lateral resolution degradation caused by inevitable physiological movements such as heartbeats during surgery. Meanwhile, our adaptive focal tracking method eliminates the need for any mechanical movement during volumetric imaging offering greater safety and stability compared to traditional surface‐tracking strategies.^[^ ^35^ ^]^ This makes it particularly well‐suited for intraoperative OC'T and angiography imaging. Although only the raster scanning protocol is used to demonstrate the effectiveness of our focus tracking method, a more intricate scanning protocol^[^ ^36^ ^]^ combined with the ETL can be employed in different situations, compensating for the limitation of ETL's response time. For instance, we can decompose a region into several long strip scans to achieve dynamic focusing along the fast axis. Additionally, our method is not restricted to any specific focal change technology, meaning that other high‐speed focal changing techniques can be applied to further enhance the dynamic focus tracking speed of FACT‐ROCT.^[^ ^37^, ^38^ ^]^

Second, the integration of a force‐controlled 7‐DOF robotic arm also enables continuous and large‐area automatic imaging, covering the entire spinal cord tumor region efficiently and safely. The robotic arm's capabilities, such as zero‐force dragging and virtual wall control, enhance safety during intraoperative imaging by preventing unintentional probe movements and collisions. By automating the movement of the OCT probe based on previously obtained volumes, the system minimizes the need for manual adjustments and reduces the risk of human error. The imaging procedure is safe and well‐tolerated, even in cases involving multiple consecutive imaging runs. No additional medications are required, and no imaging‐related side effects are observed in the participating patients. In addition, the implementation of motion compensation techniques based on fast cross‐sectional scanning addresses the deformation of OCT volumes caused by physiological movements, maintaining image integrity and reliability during surgery.

The FACT‐ROCT system is designed to integrate seamlessly into standard surgical workflows. Our intraoperative protocol involves performing an initial FACT‐ROCT scan prior to tumor resection to evaluate tumor characteristics, grade, vascular distribution, and boundary definition. This real‐time imaging information is crucial for formulating optimal resection strategies. During the resection process, surgeons conduct intermittent FACT‐ROCT scans as needed, primarily when the tumor is nearly completely resected, allowing for focused evaluations of the current vascular distribution and general tumor characteristics. With its high‐speed imaging capability of up to 280 cross‐sectional images per second, FACT‐ROCT generates structural images in ≈2.5 s and microvascular images in ≈10 s, providing near real‐time feedback without significantly prolonging surgery. Additionally, the integration of adaptive focus tracking minimizes user interference during imaging, enhancing the overall usability of the system. In terms of ergonomics, FACT‐ROCT employs a multi‐degree‐of‐freedom, force‐controlled robotic arm, allowing surgeons to maneuver the OCT probe with minimal effort, while the virtual wall feature enhances safety by ensuring precise and intuitive positioning of the probe within the surgical field. Overall, the high imaging speed and ergonomic design of FACT‐ROCT facilitate real‐time usability while effectively reducing the surgeon's burden.

In this first‐in‐human study, the FACT‐ROCT system is used for intraoperative imaging on 22 patients with various spinal cord tumors, including spinal gliomas (grades I‐IV), teratomas, hemangiomas, and lipomas, showing promising clinical outcomes. These imaging results demonstrate that the FACT‐ROCT system can provide micrometer‐scale in vivo images of normal tissues, nerve boot, and vessels, which are beneficial for spinal cord tumor surgery. Additionally, the OCT structural images from different spinal cord tumors show that FACT‐ROCT has the potential in differentiate the types of spinal cord tumors. Also, we also find high‐grade gliomas exhibited more uneven scattering, correlating with increased heterogeneity and malignancy, while low‐grade tumors showed a more even scattering pattern.

Further quantitative analysis of OAC is conducted on the different grade spinal cord gliomas. It is worth to mention that our proposed adaptive focus tracking method ensures that the focus always remains at the same position inside the tissue, which guarantees the robustness of the OAC, even in the presence of tissue movement intraoperatively. The OAC results for in situ spinal cord gliomas are significantly different from those reported for ex vivo brain gliomas in previous research,^[^ ^22^ ^]^ possibly due to factors such as the continuous blood supply in living tissues. We find it difficult to directly distinguish between different grades of tumors and normal tissue just based on the OAC, while the standard deviation of the attenuation coefficient increases with higher‐grade gliomas. Using the standard deviation of OAC as a physical marker, the system achieved 90.2%  ±  2.7% accuracy in distinguishing high‐ from low‐grade gliomas intraoperatively at a threshold of 0.75  ±  0.01 mm^−1^. This high diagnostic accuracy indicates FACT‐ROCT's potential in providing critical real‐time information for surgical decision‐making.

In addition to structural imaging, FACT‐ROCT also provides detailed vascular imaging. Unlike previous clinical studies on brain tumor vascular imaging,^[^ ^28^ ^]^ which only visualized blood vessels in B‐scan images. FACT‐ROCT, utilizing the robotic arm and the mentioned innovations, enables vascular imaging across the entire tumor area. It can cover a region of 70 mm × 13 mm × 10 mm within 2 min. This capability enables the creation of comprehensive vascular maps, essential for planning safe resections by avoiding critical blood vessels and minimizing intraoperative bleeding. Besides, our findings reveal that higher‐grade tumors exhibit greater vascular tortuosity, a marker of their aggressive nature. These observations offer valuable information for surgical planning and may also contribute to a better understanding of tumor biology.

Despite the promising results, the FACT‐ROCT system also has some limitations. First, the imaging depth of OCT is limited to 1–2 mm, which may be insufficient for larger or deeply situated tumors. However, when FACT‐ROCT seamlessly integrates into existing surgical workflows, the depth limitation becomes less of a concern, as the depth of surgical excision typically aligns with the 1–2 mm range. Additionally, bleeding during surgery can further reduce OCT imaging depth if blood covers the tissue surface, as illustrated in Figure S10 (Supporting Information). Therefore, during actual surgical procedures, we first achieve hemostasis and then rinse the area with saline to prevent blood from adversely affecting imaging depth. Furthermore, while the use of exogenous fluorophores could theoretically enhance imaging depth by providing complementary functional information, it is not suitable for OCT itself. OCT relies on the interference between incident light and reference light to generate high‐resolution structural images, whereas fluorescence signals do not interfere with the reference light and therefore do not contribute to OCT signal formation. Enhancing OCT sensitivity generally requires scattering particles as contrast agents.^[^ ^39^ ^]^ However, challenges remain regarding their effective targeting to specific regions of interest and the potential for adverse effects on human health.

Second, the integration of OCT with fluorescence molecular imaging into a multi‐modality system is another promising direction. On the one hand, fluorescence imaging can offer molecular contrast through exogenous fluorescent agents, thereby enhancing the capability for tumor boundary identification. On the other hand, NIR‐II intraoperative fluorescence imaging can provide significantly greater penetration depths.^[^ ^40^ ^]^ However, fluorescence imaging inherently lacks the depth‐resolved structural information that OCT provides. Therefore, developing such a dual‐modality system could effectively leverage the complementary strengths of both imaging modalities. Additionally, photoacoustic tomography^[^ ^41^ ^]^ has shown considerable potential for deep‐tissue vascular imaging due to its greater penetration depth and sensitivity to hemoglobin. Thus, a multimodal imaging approach combining OCT angiography with photoacoustic tomography could significantly improve the visualization of tumor vasculature.

Additionally, the proposed motion compensation method is only suited for object movement along the longitudinal axis, which is also the most common scenario during surgery, as the robotic arm can ensure that the light is approximately perpendicular to the object surface. Moreover, by employing faster MHz swept‐source lasers,^[^ ^42^ ^]^ the imaging speed of the FACT‐ROCT system can be further improved, but higher imaging speeds usually also mean lower sensitivity, lower imaging depth, and higher costs. Future designs will consider using the 10.3 MHz swept‐source laser that we previously developed.^[^ ^43^ ^]^ Additionally, this study involved a small sample size of patients with spinal cord tumors. More studies are necessary to validate these findings and assess the generalizability of FACT‐ROCT across different tumor types and patient populations. Although short‐term patient follow‐up results demonstrated the promising effectiveness of FACT‐ROCT, we acknowledge that these results may not be sufficient to confirm its long‐term benefits. Further, longitudinal studies are needed to evaluate the impact of FACT‐ROCT‐guided surgeries on long‐term patient outcomes, such as recurrence rates and functional recovery. It is also worth noting that spinal cord tumors in humans are rare, and the in vivo imaging results presented in this study are valuable clinical data, including cases such as Ewing's sarcoma and many different grades of spinal cord glioma.^[^ ^44^, ^45^ ^]^ These findings might hold significant clinical value for future spinal cord tumor surgeries.

In conclusion, the introduction of the FACT‐ROCT system represents a significant advancement in the intraoperative imaging of spinal cord tumors. By addressing critical challenges in focus tracking, motion compensation, and large‐scale scanning, our work enhances the precision and applicability of OCT in a surgical setting. The ability to perform high‐resolution structural and vascular imaging in real‐time improves the accuracy of tumor grading and provides objective, quantitative information for the timely formulation of personalized surgical strategies, thereby potentially enhancing patient outcomes and advancing the progress of “precision surgery” in spinal cord tumor treatment. Future advancements and larger studies will further elucidate its role in clinical practice and solidify its position as a vital tool in neurosurgical oncology. FACT‐ROCT also holds the potential to enhance the precision of brain–spine interface implant surgeries and expand its applications to various tumor surgeries across different departments.

## 4 Experimental Section

### Study Design

The objective of this study was to evaluate the application of a multifunctional OCT system, including both structural and vascular imaging, in imaging spinal cord tumors in patients undergoing surgical resection. Acknowledging the limitations of existing imaging technologies in providing high‐resolution, real‐time images during spinal cord tumor surgeries, the FACT‐ROCT system was developed. No additional medications were necessary for FACT‐ROCT acquisitions. To ensure the safety of the entire experiment, a non‐contact imaging approach with a high‐resolution, variable‐focus OCT probe mounted on a robotic arm, incorporating fast focus tracking and motion compensation methods was used to achieve artifact‐free imaging during surgery. The primary objective was to evaluate the system's effectiveness in providing micrometer‐resolution in vivo structural images of various spinal cord tumors and vascular imaging results, as well as its potential to enhance diagnostic accuracy and guide surgical decision‐making. The study enrolled 22 patients with spinal cord tumors, including 13 with spinal gliomas (grades I to IV) and 9 with other tumors or lesions such as teratomas. The selected tumors were all indicated by MRI to protrude from the surface of the spinal cord, and their tumor types were subsequently determined through H&E pathological examination. The OCT structural and vascular images, along with the quantitative imaging findings were presented. The OAC and vascular tortuosity of different grade spinal cord gliomas were calculated to demonstrate the increased heterogeneity with higher grade tumors and to indicate that FACT‐ROCT has the capability to serve as an intraoperative real‐time imaging method for tumor grading. All animal experiments were approved by the Institutional Animal Care and Use Committee of Tsinghua University (protocol # 24‐XP1). Participants were enrolled through a clinical study protocol approved by the Tsinghua Changgung Hospital Ethics Committee (protocol #23703‐4‐01). No randomization and blinding were performed.

### FACT‐ROCT System

The FACT‐ROCT was based on a home‐built robotic SS‐OCT system, as shown in Figure S1 (Supporting Information). A 200 kHz swept‐source laser (Axsun Technology) with a center wavelength of 1310 nm and a sweeping bandwidth of 100 nm was incorporated into the system. A 1:99 fiber coupler was used, where 1% of the light passed through a circulator into a Fiber Bragg Grating (FBG, *λ*_0_ = 1327 nm, reflectivity = 99%, ∆*λ* = 0.4 nm, Guangxin Technology) to achieve stable A‐line wavelength triggering, ensuring the phase stability of the system. The remaining 99% of the light was further split by a 10:90 coupler, with 10% going to the reference arm and 90% to the sample arm. The light was then recombined using a 50:50 coupler and directed into a 1.6 GHz balanced detector (ABD‐1.6G‐A, Wuhan Optolabs). In the sample arm, the light first passed through an ETL (EL‐10‐30‐C, Optotune), and then a 2D galvanometer (S8107, Sunny Technology) was used to achieve 2D lateral scanning. After scanning, the light passed through a scan lens (LSM04, Thorlabs) with a focal length of 54 mm, focusing on the spinal cord tumor tissue. A 632 nm red light was introduced into the sample arm to guide the scanning. The generated A‐line trigger was sent to a digital‐to‐analog (DA) card (USB 3020, ART Technology) as a clock signal, and frame triggers and galvanometer control analog signals were generated by this DA card. The interference signal was acquired by a 12‐bit acquisition card (ATS9373, AlazarTech) and then sent to a GPU for post‐processing. The real‐time OCT B‐scan signal drove a PCIe DA card (PCIe 6353, National Instruments) to generate voltage signals for ETL focal changing. The lateral scan range of the system for a single subject was 13 mm × 13 mm, and the axial scan range was 5 mm. The lateral and axial resolutions of the system were ≈25 and ≈10 µm in tissue, respectively. The time to achieve structural imaging for a single subject was 2.5 s, and vascular imaging took 10 seconds. The system achieved a sensitivity of 105 dB with a sample power of 10 mW and a sensitivity roll of is −0.2 dB per mm.

The entire sample arm was mounted on a seven‐axis force‐controlled medical robotic arm (ER3 Pro‐Med). The robotic arm had a collision detection sensitivity of less than 3 N, with a real‐time refresh rate of 1 kHz. It also supported near zero‐force lightweight dragging, making it easy for medical staff to operate. Additionally, the robotic arm was equipped with a real‐time controllable virtual wall function, which could restrict the movement range of the tool. After mounting the OCT sample arm onto the robotic arm using custom mechanical parts, a two‐step calibration method was applied to match the OCT coordinate system with the robotic arm's coordinate system,^[^ ^46^, ^47^, ^48^, ^49^ ^]^ allowing the robotic arm to move guided by the OCT images. To compensate for imaging distortion caused by heartbeat during surgery, a cross‐sectional scan was performed before volumetric imaging to obtain the surface contour of the object. This contour was used to compensate for object motion during volumetric imaging. Furthermore, after acquiring the first compensated volumetric image, the robotic arm planned the next scan position based on the last 200 frames of data from the previous volumetric scan, repeating this process to scan all areas of the spinal cord tumor (Figure S4, Supporting Information).

During OCT volumetric imaging, an adaptive focus tracking strategy based on real‐time OCT B‐scan images was used to compensate for the defocus caused by object motion and curvature. Specifically, after obtaining the first OCT B‐scan image, the surface positions of the 10 central A‐lines of the B‐scan were identified and averaged using a rapid surface detection algorithm.^[^ ^50^ ^]^ Based on the continuity of the object, the ETL was then used to adjust the focal point of the next B‐scan to ≈400 µm below the surface of the object. This method had a response time of 10 ms. By using this approach, where the previous B‐scan drove the precise focusing of the next B‐scan, almost all B‐scans of the object could be maintained in the high‐resolution region.

### Optical Attenuation Coefficient and Its Standard Deviation

The quantitative analysis of OCT structural images for different grades of spinal cord gliomas was based on their attenuation coefficients. Due to light attenuation in tissue, and considering the effects of defocusing and system fall‐off, the average OCT signal intensity could be expressed as:^[^ ^51^ ^]^

(1) $$\langle A\left(z\right)\rangle =A_{0}\times t\left(z\;-z_{f}\right)\times h\left(z\right)\times e^{\left(-\mu \times z\right)}+noise$$

where *A*_0_ is a constant, *z* is the depth pixel in OCT images, *t*(*z*  −  *z_f_*) represents the defocus‐related term. As for the adaptive focus tracking strategy, the focal point was always located 400 µm beneath the surface of the object, which was fixed to avoid inconsistencies in attenuation coefficients caused by focal changes in traditional methods. And *h*(*z*) is the sensitivity roll‐off factor term. When calculating the attenuation coefficient, regions with obvious surface blood vessel occlusion and non‐spinal cord areas at the edges were first excluded. For the remaining valid A‐lines, the surface contour of the OCT image was obtained using an automatic detection algorithm. Then, the signals below the surface from five consecutive A‐lines in the lateral direction were averaged to obtain the average light intensity 〈*A*(*z*)〉. From this dataset, several segments at equal distances below the surface were randomly selected for fitting using Equation (1) to calculate the attenuation coefficient μ. For one B‐scan, 10 A‐lines were randomly selected, resulting in 10 values of the attenuation coefficient. Then, the standard deviation of these attenuation coefficients σ_μ_ is calculated as:

(2) $$\sigma_{\mu }=\sqrt{\frac{\sum_{i=1}^{10}{\left(\mu_{i\;}-\;\bar{\mu }\right)}^{2}}{9}}$$

For each case, ≈1000 to 5000 B‐scans were collected, depending on the tumor size, representing OCT image samples from different locations of the tumor. Some B‐scans were randomly selected from this dataset, and the standard deviation of the corresponding attenuation coefficients was calculated. This standard deviation served as a statistical parameter reflecting the heterogeneity of the attenuation coefficient.

### Spinal Cord Tumor Vascular Imaging and Vessel Parameter Analysis

Label‐free vascular imaging of spinal cord tumors could be achieved by performing four consecutive B‐scans at the same location, followed by applying a vascular algorithm based on correlation‐coded eigen decomposition.^[^ ^46^, ^52^, ^53^ ^]^ To remove vertical stripe artifacts caused by intraoperative motion, a Fourier domain spatial filtering method was employed. Details of the specific vascular processing algorithm can be found in the Supporting Information (Figure S5, Supporting Information). Once the vascular imaging results were obtained, different depths of the vasculature were displayed using color mapping. To analyze the quantitative vascular parameters, the vascular results were projected into 2D enface images and vascular tortuosity,^[^ ^54^ ^]^ vascular node density, and vascular trajectory variability were selected as statistical parameters to compare the differences between high‐ and low‐grade spinal cord glioma vasculature. The average tortuosity of vessels in the region (also referred to as distance metric, DM) was defined as the total length of all vessels divided by the sum of the distances between the start and end points of all vessels. The density of vascular nodes was defined as the number of vascular branches per unit area. Vascular trajectory variability was defined as the standard deviation of the azimuth angles of the local directions of all vessels. During processing, the 2D projection of the vessels obtained from OCTA was first subjected to Gaussian filtering, Frangi filtering, and binarization. The binarized vessel map was then processed using morphological methods for denoising and subsequent extraction of the vessel skeleton, after which the vascular parameters were calculated. First, the number of vascular branches, as shown in (Figure S6A, Supporting Information), was counted from the vascular skeleton diagram and divided by the area of the region to obtain the vascular node density, defined as:

(3) $$VND=\frac{\mathrm{Number}\;\mathrm{of}\;\mathrm{nodes}}{\mathrm{Area}\;\mathrm{of}\;\mathrm{region}}$$

Next, all node positions of the vascular diagram are disconnected to generate a subgraph composed of a series of non‐intersecting vessels. For each vessel in this subgraph, the total length *L_i_* and the distance between its start and end point $s_{i}=\left|\vec{\mathrm{AB}}\right|$ are calculated, as illustrated in (Figure S6B, Supporting Information). Finally, the total lengths *L_i_* and distances *s_i_* of all vessels are summed, and the average vascular tortuosity is computed as:

(4) $$DM=\frac{\sum L_{i}}{\sum s_{i}}$$

In the subgraph composed of non‐intersecting vessels, the azimuth angles ϕ_*i*_ of each vessel at specified intervals were recorded, as shown in (Figure S6C, Supporting Information). The number of azimuth angles ϕ_*i*_ for all vessels in the region were tallied, with the most frequent ϕ_*i*_ identified as the primary flow direction ϕ_*m*_ of the vessels in that area. Then, for all azimuth angles that formed an obtuse angle with this primary direction, they are normalized to $\left(\phi_{m}-\frac{\pi }{2},\phi_{m}+\frac{\pi }{2}\right]$ by $\phi_{i}^{\prime }=\phi_{i}\pm \pi$. Subsequently, the standard deviation was calculated as the variability of vascular trajectories, expressed as:

(5)

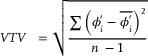

where *n* is the total number of azimuth angles involved in the statistics, and $\bar{\phi_{i}^{\prime }}$ represents the mean of all normalized $\phi_{i}^{\prime }$. From these parameters that reflect the complexity of the vessels, high‐grade tumors with complex and disordered vascular structures could be distinguished from low‐grade tumors with simpler and more organized vascular patterns.

### Statistical Analysis

In order to determine the required sample size for the study, a power analysis was conducted. The effect size, calculated from pre‐experiments, was 1.868. Under the requirements of significance level *α* = 0.05, statistical power 0.8, and a one‐tailed *t‐*test power analysis, a minimum of 5 patients per group was required. During the analysis, a significance test was performed to evaluate the difference in the standard deviation of the attenuation coefficient (σ_μ_) between low‐grade and high‐grade spinal cord gliomas. A one‐tailed *t*‐test was used to assess whether the difference in σ_μ_ between the two groups was statistically significant. Representative data from 13 patients with WHO Grade I, II, III, and IV gliomas were selected for analysis. From the thousands of B‐scans obtained from each patient, over 10 B‐scans containing tumor regions (≈1% of the total B‐scans, at least 1 mm apart) were randomly selected for the calculation of σ_μ_. The low‐grade and high‐grade groups each contained ≈120 σ_μ_ values. The sample size satisfied the assumptions for conducting a one‐tailed *t*‐test, providing a statistical power of greater than 0.8 with a significance level of *α* = 0.05, indicating a statistically significant difference in the standard deviation of the attenuation coefficient (σ_μ_) between the low‐grade and high‐grade tumors. For the classification accuracy metrics and their dependent OAC variability threshold, leave‐one‐out cross‐validation was performed. The testing result is shown in Figure S9 (Supporting Information).

To assess the differences in the three vascular parameters between the low‐grade and high‐grade groups, a one‐tailed *t*‐test was also conducted. Each sample was obtained from a randomly selected subregion of the tumor area. On average, each case was divided into five subregions, and the parameters were calculated separately for each. Approximately 25 subregions were analyzed for both the low‐grade and high‐grade groups. The sample size also met the requirements for performing a one‐tailed *t*‐test with a significance level of *α* = 0.05 and a statistical power of 0.8. The *t*‐test results yielded *p*‐values of 0.006, 0.006, and 0.004 for the three vascular parameters, respectively, confirming statistically significant differences between the low‐grade and high‐grade groups for all three parameters. The power analysis was completed using G*Power software, and subsequent statistics were performed using MATLAB.

## Conflict of Interest

The authors declare no conflict of interest.

## Supporting information



Supporting Information

## Data Availability

The data that support the findings of this study are available from the corresponding author upon reasonable request.
