# Supplementary material for: Intraoperative Fast Adaptive Focus Tracking Robotic OCT Enables Real‐Time Tumor Grading and Large‐Area Microvascular Imaging in Human Spinal Cord Surgery
Source: Adv Sci (Weinh). 2025 Apr 25;12(27):2503566. doi: 10.1002/advs.202503566 (PMC12279235; doi:10.1002/advs.202503566)
Supplement: Supplementary file 1 — Supporting Information [file ADVS-12-2503566-s001.docx]

**Supporting Information**

Intraoperative Fast Adaptive Focus Tracking Robotic OCT Enables Real-Time Tumor Grading and Large-area Microvascular Imaging in Human Spinal Cord Surgery

Bin He, Yuzhe Ying, Yejiong Shi, Zhe Meng, Zichen Yin, Zhengyu Chen, Zhangwei Hu, Ruizhi Xue, Linkai Jing, Yang Lu, Zhenxing Sun, Weitao Man, Youtu Wu, Dan Lei, Ning Zhang, Guihuai Wang* and Ping Xue*


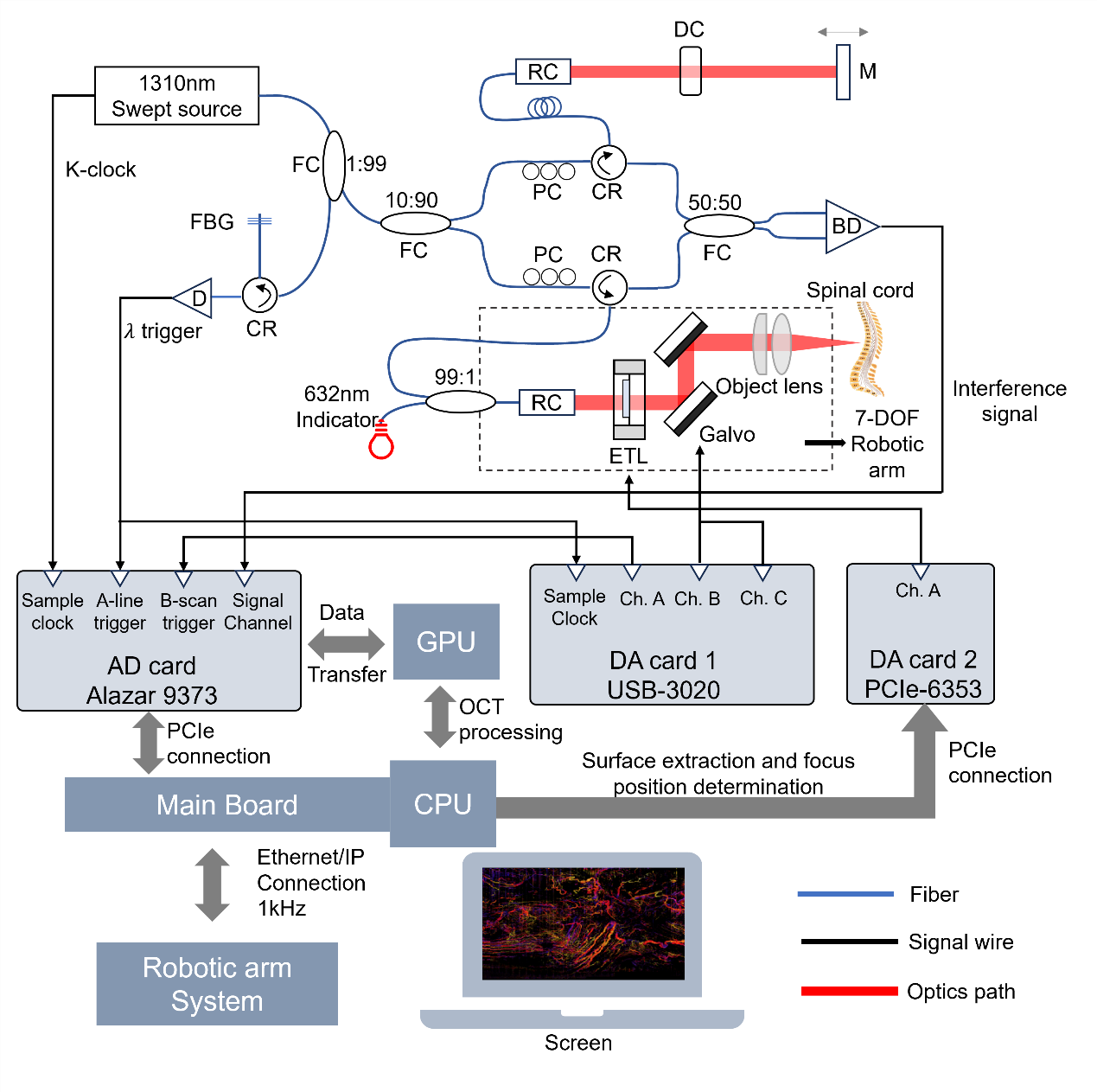


**Figure S1. Schematic of FACT-ROCT system**. Abbreviations: FC, fiber coupler; FBG, fiber bragg grating; ETL: electrically tunable lens; RC, reflective collimator; PC, polarization controller; BD: balanced detector.

**
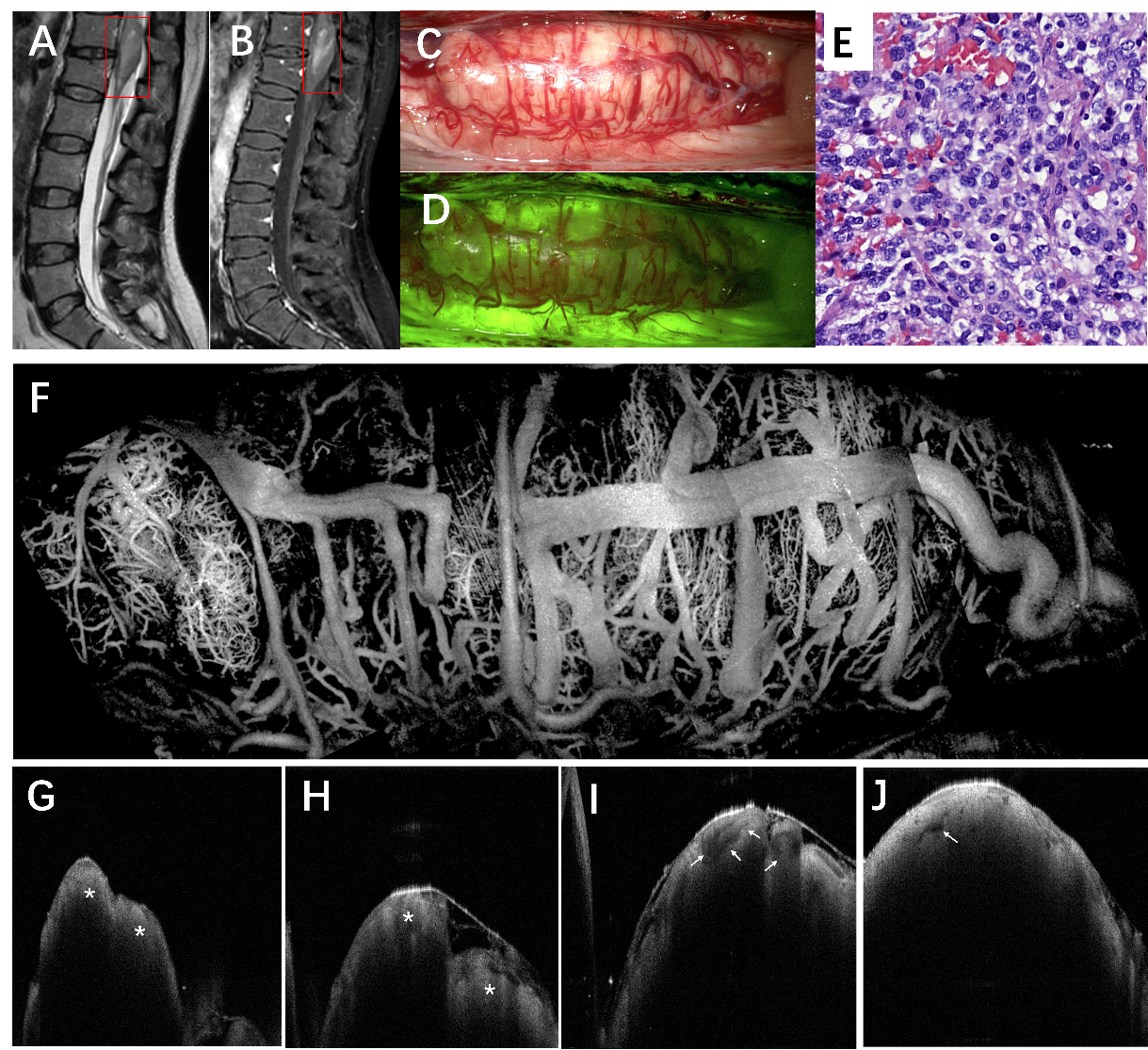
**

**Figure S2. A case of diffuse midline glioma:** (A-B) Preoperative MRI of the patient shows the tumor located at the T12-L1 vertebral level, occupying most of the spinal cord, causing fusiform swelling and surface breakthrough. Contrast-enhanced MRI shows significant and also heterogeneous signals. (C) Intraoperative macroscopic view of the tumor shows that the tumor is prominently protruding from the surface of the spinal cord, with a rich blood supply and a high density of surface vessels. (D) Intraoperative fluorescence imaging of the tumor after sodium fluorescein injection shows fluorescein enrichment on the left side of the tumor. (E) Postoperative pathology image of the tumor specimen shows densely distributed tumor cells of various shapes, with some cells displaying a plump spindle shape and marked atypia. Microvascular proliferation and hemorrhage are also observed, characteristic of WHO Grade IV glioma. (F) OCTA vascular imaging shows dense and irregular tumor vasculature, with large surface vessels corresponding to the macroscopic image in panel C. (G-J) Selected OCT B-scan structural images from the tumor’s rostral to caudal ends show heterogeneous optical signals within the tumor (indicated by "*"), with some vessels traversing the tumor surface or penetrating through the tumor (as indicated by white arrows).

**
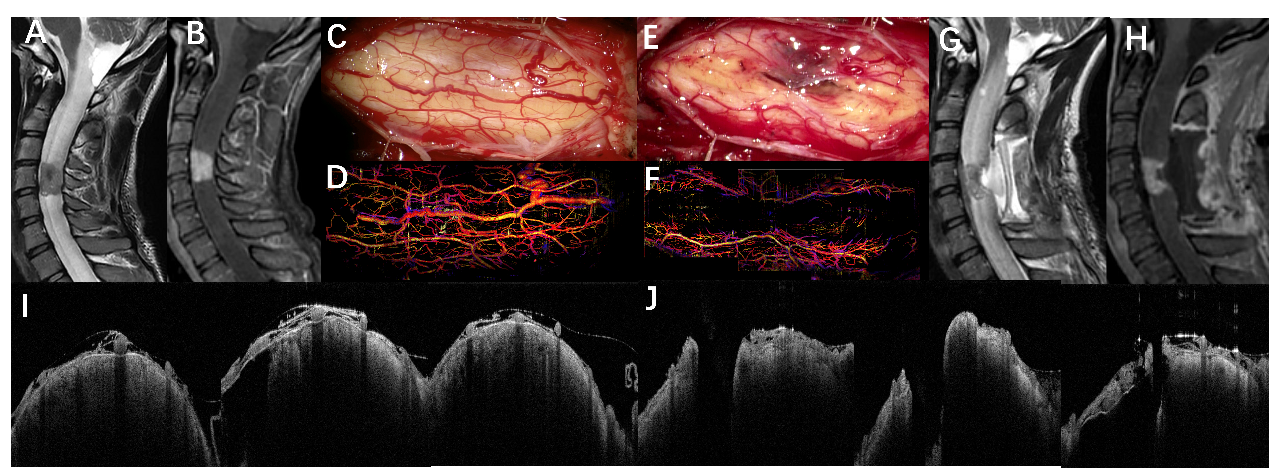
**

**Figure S3. A case of subependymal tumor:** (A-B) Preoperative MRI shows the tumor located within the cervical spinal canal, with a clearly enhanced lesion that has broken through the spinal cord surface. (C) Intraoperative macroscopic view of the tumor shows the tumor surface as light grayish-red, located on the right side of the tumor. The surface vasculature appears sparse and not significantly disorganized. (D) OCTA vascular imaging shows sparse vasculature with no apparent disorganization, with some surface vessels corresponding to the macroscopic view in panel C. (E) Macroscopic view of the surgical cavity after tumor resection and pia mater suturing. (F) OCTA vascular image after tumor resection and pia mater suturing shows moderate vascular density on the spinal cord surface, with no vascular signals inside the surgical area, corresponding to the macroscopic view in panel E. (G-H) Postoperative MRI shows residual tumor following resection, with no hematoma or fluid accumulation in the surgical area. (I) Selected OCT B-scan structural images from the rostral to caudal ends of the tumor show heterogeneous signals within the tumor, with calcification and cystic degeneration signals present. (J) Selected OCT B-scan structural images post-tumor resection show gaps after pia mater suturing, with optical signals similar to those observed before tumor resection, suggesting residual tumor, corresponding to panels G-H.


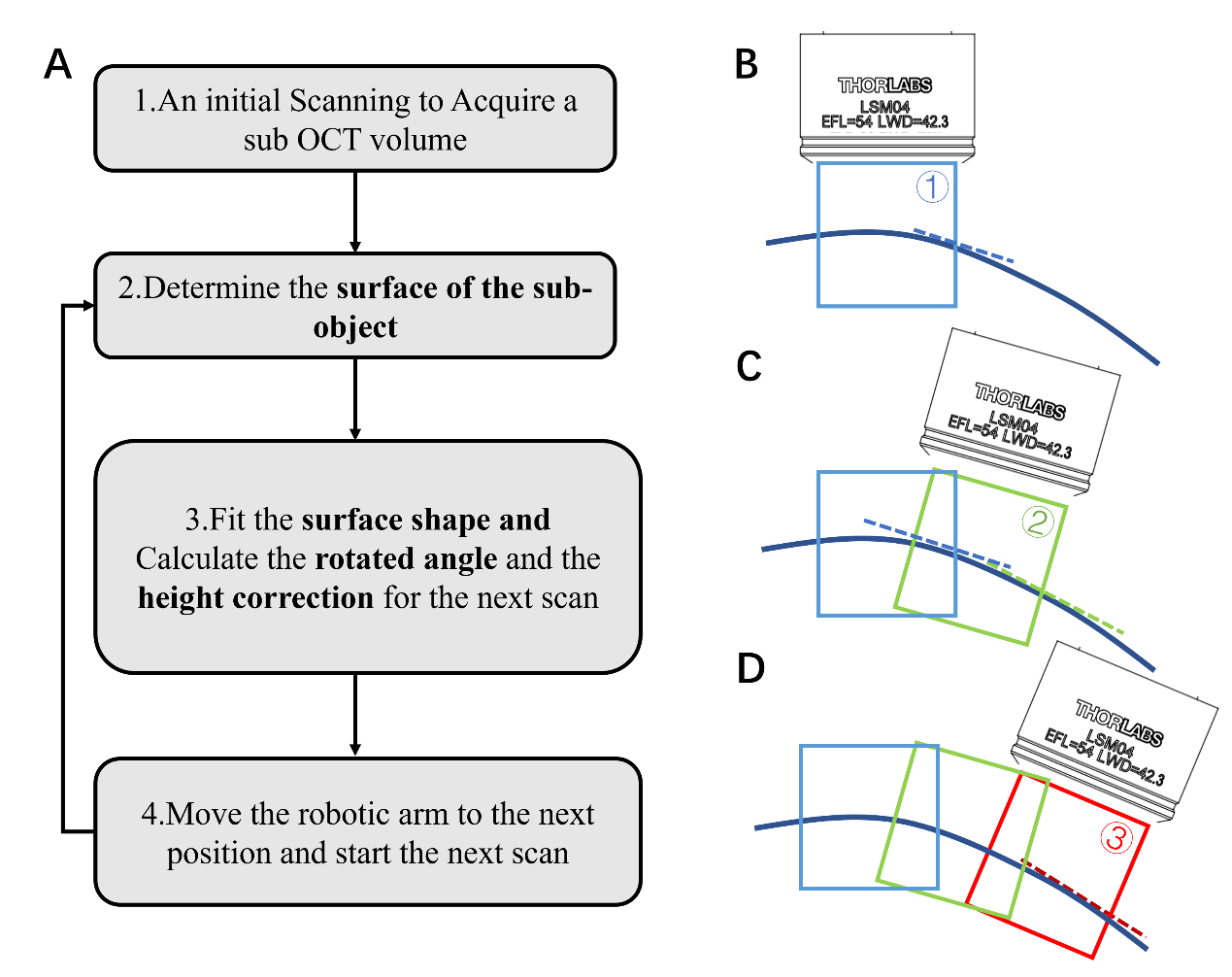


**Figure S4. Automatic large-scale scanning mechanism employed by the FACT-ROCT system:** (A) Flowchart of the automatic large-scale scanning process. The core component involves the movement of the robotic arm driven by the previous OCT volume data to achieve extensive scanning. (B)-(D) Schematic diagrams illustrating three consecutive OCT volume scans.


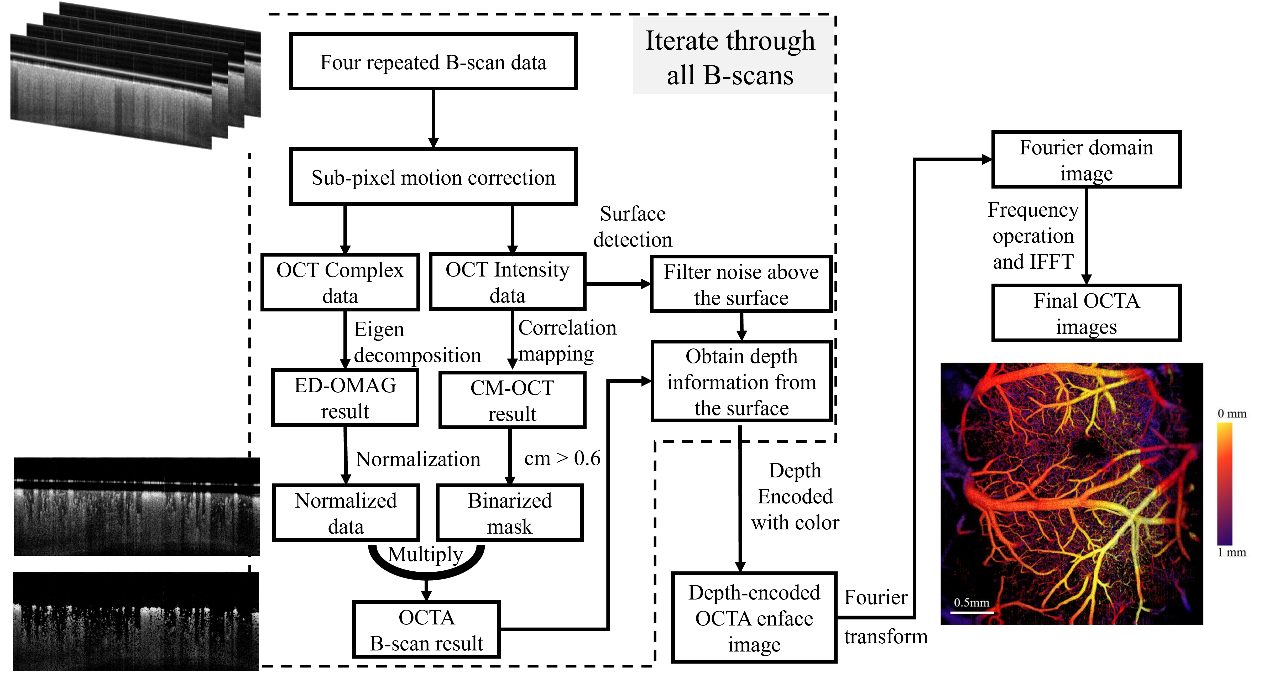


**Figure S5. The flow chart of OCTA algorithm:** The process begins with four repeated B-scan data, followed by sub-pixel motion correction and surface detection. OCT complex data undergo eigen decomposition, yielding eigen decomposition optical microangiography (ED-OMAG) results, while intensity data are processed through correlation mapping to generate correlation mapping OCT (CM-OCT) results. After normalization and binarization, the OCTA B-scan results are obtained. Surface depth information is retrieved and encoded with color. The depth-encoded OCTA enface image is then subjected to a Fourier transform, resulting in the final OCTA images through frequency operation and inverse Fourier transform (IFFT). The final image on the right shows a depth-encoded vascular map in an enface OCTA image, with color representing different depths.


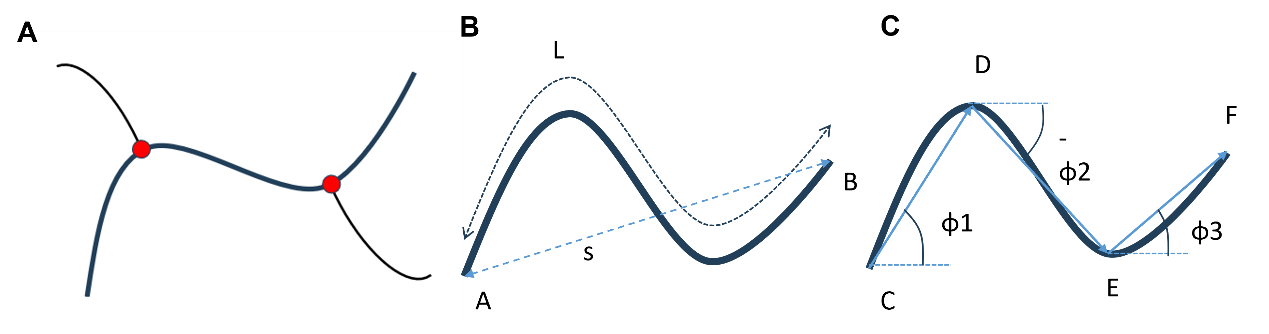


**Figure S6. Illustration of vascular parameter analysis calculations:** (A) Vascular branch points indicated by red dots. (B) The definition of vascular tortuosity for a single vessel. (C) The orientation azimuth statistics represent the trajectory variability of a single vessel.


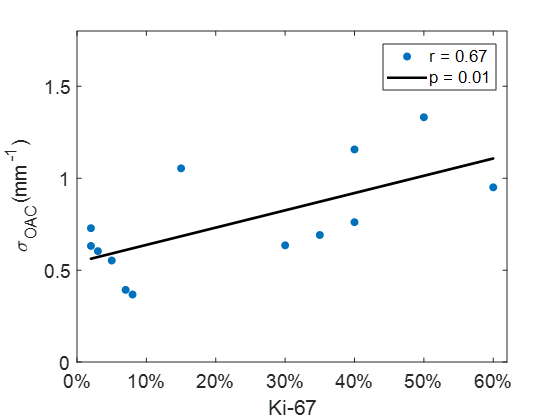


**Figure S7. Positive correlation between OAC variability and Ki-67 index.** Each point represents the average data of OAC variability and Ki-67 index from an individual glioma patient included in this study (n = 13). The analysis reveals a correlation coefficient of r = 0.67 and a p-value of 0.01, indicating a significant positive relationship.


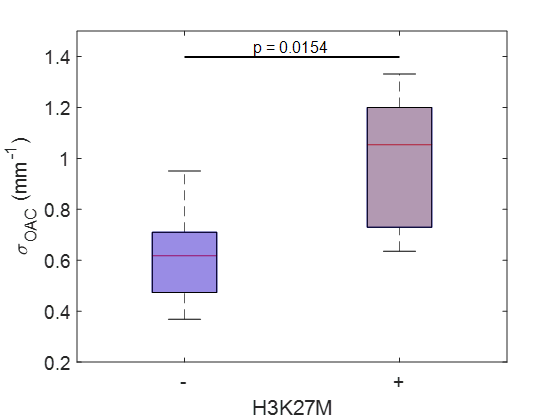


**Figure S8. Boxplot comparison of OAC variability in glioma patients based on H3K27M status.** The analysis (n = 13) reveals significantly higher OAC variability in the positive group (p = 0.0154).


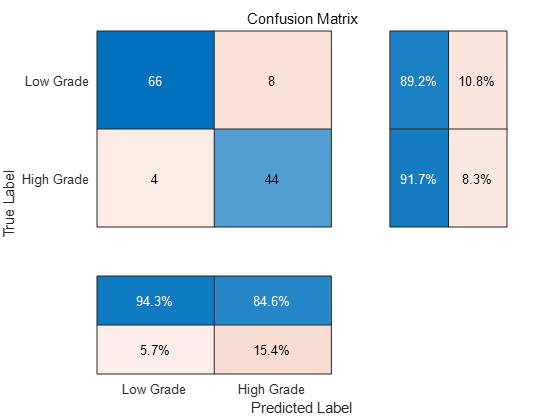


**Figure S9. Confusion Matrix of Accuracy for Glioma Grading Using OAC Varieties Extracted from Different B-scans (n = 122) with Leave-One-Out Method.** The top-left diagram shows the confusion matrix, where rows represent True Labels and columns represent Predicted Labels. For Low Grade samples, 66 are correctly classified (89.2%), while 8 are misclassified as High Grade (10.8%). For High Grade samples, 44 are correctly classified (91.7%), while 4 are misclassified as Low Grade (8.3%). The top-right diagram displays the Precision for each class, with 89.2% for Low Grade samples and 91.7% for High Grade samples. The bottom diagram shows the Recall for each class, with 94.3% for Low Grade samples and 84.6% for High Grade samples.


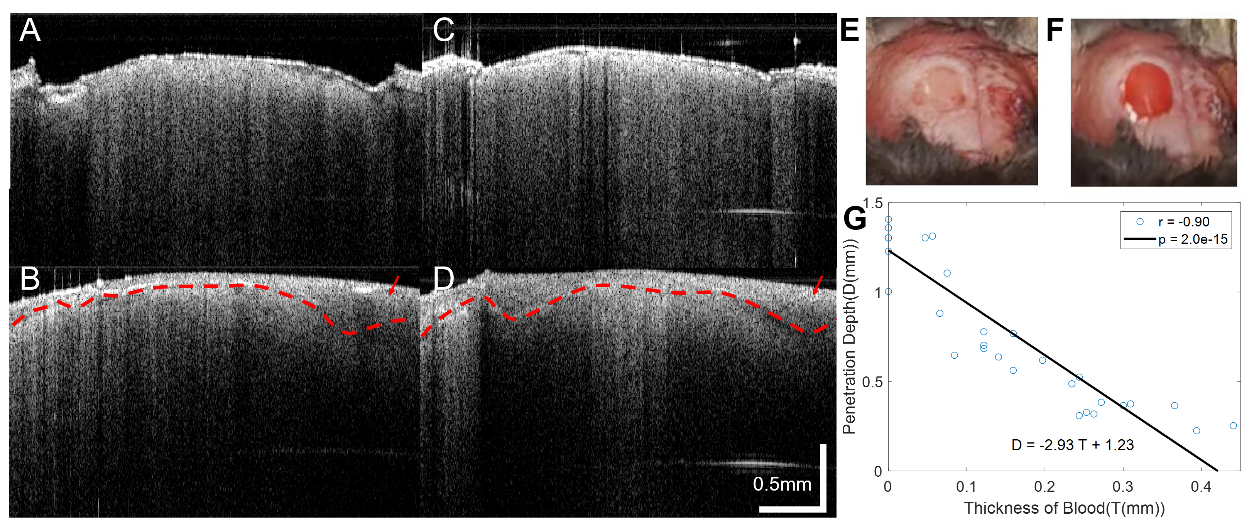


**Figure S10. Imaging results from the mouse simulated hemorrhage experiment.** (A) (C) The B-scan results of OCT at two locations without hemorrhage; (B) (D) The corresponding B-scan results of OCT under simulated hemorrhage at locations A and C; The red dashed line represents the boundary between blood and tissue. To better visualize the boundary, it has been slightly shifted downward. (E) A photograph of the mouse brain without hemorrhage; (F) A photograph of the mouse brain under simulated hemorrhage; (G) The specific blood thickness from 30 different locations and fits the relationship between OCT penetration depth and surface blood thickness, with the fitted relationship given by $D=-2.93T+1.23$, $p=2.0\times{10}^{-15}$. This indicates that under conditions without surface hemorrhage, the OCT system achieves a penetration depth of approximately 1.2 mm. When the blood accumulation reaches 80 μm, the penetration depth decreases to about 1 mm; when the blood accumulation reaches 250 μm, the penetration depth further decreases to approximately 0.5 mm. The presence of blood at the target surface significantly increases light scattering, leading to an attenuation that is approximately three times greater than that of the surrounding tissue, which can consequently impact imaging quality. Therefore, it is necessary to timely clear any surface hemorrhage before imaging during surgery to ensure the quality of the images.

**Table S1**

**Comparison of FACT-ROCT with Common Intraoperative Imaging Modalities**

| Modality | Spatial Resolution | Imaging Depth | Exogenous Label? | Intraoperative Real-time? | Key Clinical Use |
| --- | --- | --- | --- | --- | --- |
| MRI | ~0.5–1 mm | Whole body | Typically no (contrast optional) | No | Large-scale anatomical imaging; limited utility for real-time surgical guidance due to low speed. |
| CT | ~0.5–1 mm | Whole body | Typically no (contrast optional) | Limited real-time use in hybrid OR setups | Bony structures, implant positioning, and broad anatomical overviews; not ideal for microstructure. |
| Ultrasound | ~100–300 µm | several cm | No | Yes | Contact measurement required. operator-dependent, relatively lower resolution. |
| Intraoperative Fluorescence Imaging | ~50–200 µm (depends on imaging system) | Lacks depth-resolved imaging capability | Yes (e.g., ICG or other dyes) | Yes | Highlights perfusion or tumor-associated markers; requires injection of contrast agents. |
| Confocal Endomicroscopy | ~1 µm lateral, ~5 µm axial | Typically  ~100–200 µm | Yes (fluorescent dyes required) | Yes | Cellular-level imaging of tissue microarchitecture; shallow depth, small field of view. |
| FACT-ROCT (This Work) | ~10 µm axial, ~25 µm lateral | Typically  1–2 mm | No | Yes (rapid adaptive focus tracking) | Real-time, label-free, high-resolution imaging of tumor microstructure and vasculature; ~10 ms focus adjustment. |

**Table S2**

**The basal information of patients cohort**

| # | Age at surgery | Sex | Diagnosis | Recurrence(Y/N) | Tumor location | Immunohistochemistry | | | |
| --- | --- | --- | --- | --- | --- | --- | --- | --- | --- |
|  |  |  |  |  |  | **GFAP(+/-)** | **Ki-67(%)** | **CD34(+/-)** | **H3K27M(+/-)** |
| 1 | 11 | F | Ganglioglioma (WHO1) | N | Medulla oblongata -C3 | **+** | **5%** | **+** | **-** |
| 2 | 33 | F | Subependymoma (WHO1) | N | C4-C5 | **+** | **2%** | **-** | **-** |
| 3 | 13 | M | Pilocytic myxoid astrocytoma (WHO2) | Y | Medulla oblongata -T10 | **+** | **3%** | **-** | **-** |
| 4 | 52 | M | Diffuse astrocytoma (WHO2) | N | C2-C4 | **+** | **8%** | **-** | **-** |
| 5 | 29 | F | Ependymoma (WHO2) | N | Medulla oblongata -C2 | **+** | **2%** | **+** | **-** |
| 6 | 33 | M | Ependymoma (WHO2) | N | C6-T1 | **+** | **7%** | **-** | **-** |
| 7 | 26 | M | Anaplastic astrocytoma (WHO3) | N | T4-T7 | **+** | **15%** | **-** | **+** |
| 8 | 4 | M | Anaplastic ependymoma (WHO3) | Y | C1-C7 | **+** | **60%** | **-** | **-** |
| 9 | 18 | F | Anaplastic ganglioglioma (WHO3) | N | Medulla oblongata -C3 | **+** | **35%** | **-** | **-** |
| 10 | 54 | M | Diffuse midline glioma (WHO4) | Y | C2-C6 | **+** | **30%** | **-** | **+** |
| 11 | 56 | M | Diffuse midline glioma (WHO4) | N | T11-T12 | **+** | **50%** | **-** | **+** |
| 12 | 40 | F | Diffuse midline glioma (WHO4) | N | T11-T12 | **+** | **40%** | **-** | **+** |
| 13 | 13 | M | Diffuse midline glioma (WHO4) | N | T4-T7 | **+** | **40%** | **-** | **+** |
| 14 | 55 | F | Angioreticuloma | N | Medulla oblongata | **-** | **4%** | **+** | **-** |
| 15 | 41 | F | Teratoma | Y | T11-L2 | **-** | **＜1%** | **-** | **-** |
| 16 | 9 | F | Myelitis | N | C3-C4 | **+** | **＜1%** | **+** | **-** |
| 17 | 41 | M | Epidermoid cyst | N | T12-L1 | **-** | **1%** | **-** | **-** |
| 18 | 21 | F | Metastatic malignant meningioma | Y | T11-T12 | **-** | **70%** | **-** | **-** |
| 19 | 10 | F | Ewing sarcoma | N | Medulla oblongata -C2 | **-** | **40%** | **-** | **-** |
| 20 | 71 | M | Spinal arteriovenous fistula | N | T9-T10 | **-** | **1%** | **-** | **-** |
| 21 | 30 | F | VHL syndrome | N | C4-T1 | **-** | **5%** | **+** | **-** |
| 22 | 18 | M | Cavernous hemangioma | N | C1-C2 | **-** | **＜1%** | **+** | **-** |

**Table legend: GFAP:** glial fibrillary acidic protein. **Ki-67:** a protein associated with cell proliferation and is widely used as a marker to measure cell growth activity in tumors or other diseases. **CD34:** a cell surface glycoprotein that acts primarily as a marker for vascular endothelial cells. **H3K27M:** a specific mutation in the histone H3 gene that is associated with certain highly malignant human brain gliomas in children and adults. This mutation leads to abnormal epigenetic regulation, promotes tumorigenesis, and serves as an important molecular marker for diagnosis and prognosis. **F:** female; **M:** male; **Y:** yes; **N:** no; **“+”**: positive; **“-”**: negative.

**TableS3**

**The information of patients’ clinical date**

| # | Age at surgery | Sex | Preoperative symptoms | | | | Operation | | | Postoperative day 7 symptoms | | | | Follow-up | |
| --- | --- | --- | --- | --- | --- | --- | --- | --- | --- | --- | --- | --- | --- | --- | --- |
|  |  |  | **Motor function** | **Sensory function** | **Bowel and bladder function** | **Other symptoms** | **operation time（hours）** | **Volume of bleeding in operation（ml）** | **EOR（GTR/STR/PR）** | **Postoperative motor function** | **Postoperative sensory function** | **Postoperative bowel and bladder function** | **Other symptoms** | **Follow-up time（months）** | **Result of follow-up** |
| 1 | 11 | F |  |  |  | Vomiting and Choking on water | 7.2 | 50 | STR | Muscle strength in all four limbs: grade 3 |  |  | Decreased spontaneous respiratory function | 12 | Normal spontaneous respiration with grade 4 muscle strength in all four limbs |
| 2 | 33 | F | Right-sided muscle strength: grade 4 | Limb numbness (all four limbs) |  |  | 5.03 | 50 | PR | Right-sided muscle strength: grade 4 | Alleviation of numbness in all four limbs |  |  | 10 | Right-sided muscle strength: grade 4 |
| 3 | 13 | M | Bilateral upper limbs strength: grade 4 |  |  | Headache | 15.35 | 400 | GTR | Bilateral upper limb muscle strength: grade 4 |  |  | Alleviation of headache | 12 | No symptoms |
| 4 | 52 | M |  | Numbness in the right fingertips |  | Neck pain | 5.66 | 100 | GTR | Muscle strength in all four limbs: grade 5 | Resolution of right fingertip numbness |  |  | 16 | No symptoms |
| 5 | 29 | F | Right-sided muscle strength: grade 3 |  |  |  | 5.66 | 100 | GTR | Right-sided muscle strength: grade 3 |  |  |  | 12 | Right-sided muscle strength: grade 4 |
| 6 | 33 | M |  | Left cervicoscapular and upper extremity pain |  |  | 3.60 | 50 | GTR |  | Pain relief |  |  | 10 | No symptoms |
| 7 | 26 | M |  | Decreased pain and temperature sensation in the left lower limb |  |  | 5.08 | 200 | GTR | Muscle strength in all four limbs: grade 4 | Decreased pain and temperature sensation in the left lower limb |  |  | 12 | Right lower limb muscle strength grade 4 with grade 5 in all other extremities |
| 8 | 4 | M |  | Bilateral shoulder pain |  |  | 5.70 | 100 | GTR |  | Pain relief |  |  | 10 | Mild shoulder pain |
| 9 | 18 | F |  | Numbness in the right fingertips |  | Neck pain | 6.47 | 100 | GTR | Right-sided muscle strength: grade 3 |  |  |  | 8 | Right-sided muscle strength: grade 4 |
| 10 | 54 | M | Bilateral upper limb muscle strength: grade 3 |  |  |  | 7.55 | 200 | STR | Muscle strength in all four limbs: grade 3 |  |  |  | 8 | Tumor recurrence, refractory to chemoradiotherapy, resulting in death |
| 11 | 56 | M | Left lower limb muscle strength: grade 4 | Numbness in both lower limbs |  |  | 4.93 | 50 | GTR | Left lower limb muscle strength grade 4 and right grade 3 | Alleviation of numbness in both lower limbs |  |  | 16 | Bilateral lower limb muscle strength grade 4 |
| 12 | 40 | F | Bilateral lower limb muscle strength: grade 2 |  | Altered rectal and urinary sensation |  | 5.28 | 200 | GTR | Bilateral lower limb muscle strength grade 2 |  | Improvement in bowel and bladder sensory dysfunction compared to preoperative status |  | 15 | Bilateral lower limb muscle strength grade 3 |
| 13 | 13 | M | Bilateral lower limb muscle strength: grade 0 | Numbness in both lower limbs | Bowel and bladder incontinence |  | 5.08 | 100 | GTR | Bilateral lower limb muscle strength grade 0 | Alleviation of numbness in both lower limbs | Improvement in bowel and bladder incontinence compared to preoperative status |  | 8 | Bilateral lower limb muscle strength grade 3 |
| 14 | 55 | F |  |  |  | Dizziness | 4.45 | 50 | GTR |  |  |  | Dizziness relief | 15 | No symptoms |
| 15 | 41 | F | Right lower limb muscle strength: grade 2 |  | Bowel and bladder incontinence |  | 5.87 | 100 | GTR | Right lower limb muscle strength grade 3 |  | Improvement in bowel and bladder incontinence compared to preoperative status |  | 13 | Right lower limb muscle strength grade 3 |
| 16 | 9 | F | Right-sided muscle strength: grade 3 | Right shoulder pain |  |  | 2.70 | 20 | GTR | Right-sided muscle strength grade 3 |  |  |  | 10 | Right-sided muscle strength: grade 4 |
| 17 | 41 | M | Bilateral lower limb muscle strength: grade 4 | Numbness in both lower limbs | Bowel and bladder incontinence |  | 6.45 | 200 | GTR | Bilateral lower limb muscle strength grade 3 |  | Improvement in bowel and bladder incontinence compared to preoperative status |  | 10 | Bilateral lower limb muscle strength grade 4 |
| 18 | 21 | F | Bilateral lower limb muscle strength: grade 1 |  |  |  | 3.90 | 100 | GTR | Bilateral lower limb muscle strength grade 1 |  |  |  | 4 | Death due to spinal canal metastases from malignant meningioma |
| 19 | 10 | F |  |  |  | Posterior cervical pain | 4.52 | 50 | GTR | Bilateral lower limb muscle strength grade 2 |  |  | Relief of posterior cervical pain | 8 | Bilateral lower limb muscle strength grade 4 |
| 20 | 71 | M |  |  |  | Lumbar pain | 6.07 | 200 | STR |  |  |  | Lumbar pain relief | 12 | No symptoms |
| 21 | 30 | F | Bilateral upper limb muscle strength: grade 4 and lower limb: grade 1 | Numbness in both lower limbs |  |  | 5.58 | 150 | GTR | Bilateral upper limb muscle strength: grade 4 and lower limb: grade 2 | Alleviation of numbness in both lower limbs |  |  | 4 | Bilateral upper limb muscle strength grade 4 and lower limbs grade 3 |
| 22 | 18 | M |  | Numbness in the left upper limb |  |  | 4.15 | 100 | GTR |  | Alleviation of numbness in the left upper limb |  |  | 6 | No symptoms |

**Table legend:** The muscle strength grading scale (0–5) used in this study was established by the British Medical Research Council (MRC) to quantify the degree of muscle strength impairment in patients with neurological or muscular disorders. The core criteria are as follows: Grade 0: No muscle contraction is detected; Grade 1: Slight muscle contraction is palpable or observable, but insufficient to produce joint movement; Grade 2: The limb can move horizontally along a surface but cannot overcome gravity; Grade 3: The limb can complete full range of motion against gravity but cannot resist additional resistance; Grade 4: The limb can overcome gravity and partial resistance, though with reduced strength compared to normal; Grade 5: Normal muscle strength, with full ability to resist resistance. According to the 72-hour postoperative MRI, extent of resection (EOR) classified as: gross total resection (GTR), subtotal resection (STR, <20% residual tumor volume), or partial resection (PR, ≥20% residual). F: female; M: male.
